# Positive Effects of Crop Diversity on Productivity Driven by Changes in Soil Microbial Composition

**DOI:** 10.3389/fmicb.2021.660749

**Published:** 2021-04-15

**Authors:** Laura Stefan, Martin Hartmann, Nadine Engbersen, Johan Six, Christian Schöb

**Affiliations:** Institute of Agricultural Sciences, Department of Environmental Systems Science, ETH Zürich, Zurich, Switzerland

**Keywords:** intercropping, soil microbial communities, biodiversity–productivity relationship, sustainable agriculture, annual crop yield, crop diversification

## Abstract

Intensive agriculture has major negative impacts on ecosystem diversity and functioning, including that of soils. The associated reduction of soil biodiversity and essential soil functions, such as nutrient cycling, can restrict plant growth and crop yield. By increasing plant diversity in agricultural systems, intercropping could be a promising way to foster soil microbial diversity and functioning. However, plant–microbe interactions and the extent to which they influence crop yield under field conditions are still poorly understood. In this study, we performed an extensive intercropping experiment using eight crop species and 40 different crop mixtures to investigate how crop diversity affects soil microbial diversity and activity, and whether these changes subsequently affect crop yield. Experiments were carried out in mesocosms under natural conditions in Switzerland and in Spain, two countries with drastically different soils and climate, and our crop communities included either one, two or four species. We sampled and sequenced soil microbial DNA to assess soil microbial diversity, and measured soil basal respiration as a proxy for soil activity. Results indicate that in Switzerland, increasing crop diversity led to shifts in soil microbial community composition, and in particular to an increase of several plant-growth promoting microbes, such as members of the bacterial phylum *Actinobacteria*. These shifts in community composition subsequently led to a 15 and 35% increase in crop yield in 2 and 4-species mixtures, respectively. This suggests that the positive effects of crop diversity on crop productivity can partially be explained by changes in soil microbial composition. However, the effects of crop diversity on soil microbes were relatively small compared to the effects of abiotic factors such as fertilization (three times larger) or soil moisture (three times larger). Furthermore, these processes were context-dependent: in Spain, where resources were limited, soil microbial communities did not respond to crop diversity, and their effect on crop yield was less strong. This research highlights the potential beneficial role of soil microbial communities in intercropping systems, while also reflecting on the relative importance of crop diversity compared to abiotic drivers of microbiomes and emphasizing the context-dependence of crop–microbe relationships.

## Introduction

The past century has seen the emergence and the development of modern, intensive agriculture that was accompanied by a strong increase in productivity per unit land area (Barrios, 2007). However, the negative effects associated with this intensification now call for a transformation toward more sustainable agricultural production (Tilman et al., 2011). Current intensive agriculture indeed has negative effects on biodiversity (Newbold et al., 2015) and ecosystem functioning (Barrios, 2007), including that of soils (Gardi et al., 2013). In particular, chemical inputs and the loss of crop diversity associated with intensive agriculture are known to negatively affect soil biodiversity (Cavicchioli et al., 2019), with consequences on soil ecosystem functioning, such as nutrient cycling, carbon storage, soil structure regulation, and pest and disease control (Wall and Bardgett, 2012; Wagg et al., 2014; Fanin et al., 2017). Losses of these essential soil functions can restrict plant growth and subsequently crop yield (Giller, 2001; Bardgett and Van Der Putten, 2014).

Numerous studies demonstrated a strong link between soil microbes and plants (Wardle et al., 2004; Fry et al., 2018). First, direct specific linkages between a plant species and a particular type of microbe, such as symbiotic interactions, might occur (Wubs and Bezemer, 2016). Moreover, plants have the ability to alter the soil chemical and physical conditions (López-Angulo et al., 2020). Some of these changes can be due to above-ground mechanisms, such as litter inputs or variations in microclimate associated with plant canopy cover (Maestre et al., 2009; Trinder et al., 2009; Delgado-Baquerizo et al., 2018). Others may be the result of below-ground processes, such as increases in soil loosening and aeration due to root growth, release of root exudates or plant signaling molecules, and changes in ion uptake (Doornbos et al., 2012; Eisenhauer et al., 2017). Therefore, a greater richness of plants, and subsequently, roots, would lead to a greater diversity of microhabitats, particularly in the rhizosphere (Philippot et al., 2013). The resulting microhabitat heterogeneity would then further enhance microbial diversity (Wardle, 2006; Eisenhauer et al., 2010), which could also increase soil functioning, following the diversity-ecosystem functioning relationship (Tilman et al., 2006). For instance, Steinauer et al. (2015) showed that increasing plant diversity led to a significant increase in soil microbial biomass and enzymatic activity. Similarly, results from a grassland biodiversity experiment demonstrated that higher plant diversity resulted in increased microbial activity and carbon storage (Lange et al., 2015). Most of these studies were performed in natural grasslands; however, the extent to which the same effects would arise in diversified cropping systems is still unclear. Indeed, intercropping, which is defined as growing at least two crops at the same time on the same field, could be a promising way to enhance soil microbial diversity and functioning by increasing plant diversity in arable systems. This increase in soil diversity and functioning could then feedback on crop yield through enhanced microbial activity and nutrient mobilization or decreased pathogen accumulation (Zhao et al., 2011; Bender and van der Heijden, 2015; Wang et al., 2017).

However, despite current technological advances to study soil microbes, soil microbial communities and their assembly mechanisms are still poorly understood (Van der Putten et al., 2013; López-Angulo et al., 2020). In particular, we are still unsure what soil microbes respond to—e.g., plant aboveground traits, plant root traits, plant community composition, plant functional diversity—and if these responses to the biotic environment are comparable to the effects of abiotic conditions, such as soil moisture, or nutrients. Consequently, the extent to which plant–microbe interactions in soils could influence crop yield of intercropping systems is still unclear. To shed light on this subject, we conducted an extensive common garden experiment including eight annual crop species belonging to four functional groups, and 40 different crop mixtures of two and four species, in which we determined soil microbial composition and measured soil respiration—as a proxy for soil functioning—in addition to crop yield. Moreover, we repeated the mesocosm experiment in two different countries—i.e., in Spain and Switzerland, which differ drastically in terms of climate and soil, and with and without fertilizer application in a fully factorial design. This experimental setup allowed us to examine the following research questions: (1) How does crop diversity affect soil microbial diversity and respiration in comparison to abiotic properties? (2) Are changes in soil microbial diversity and respiration related to crop yield? (3) Are crop–soil microbe relationships and their effects on crop yield environmentally context-dependent? We hypothesized that increasing crop diversity would lead to an increase in microbial (alpha) diversity as well as changes in bacterial and fungal community compositions—e.g., increases in symbionts, decomposers, or disease suppressive microbes—and that these changes would have a positive effect on soil basal respiration and crop yield. Finally, we hypothesized that changes in crop–microbe relationships and their impacts on crop yield would be context-dependent, with varying size effects in Switzerland and in Spain, which differ in terms of resource availability and environmental harshness.

## Materials and Methods

### Study Sites

The crop diversity experiment took place in outdoor experimental gardens in Zurich, Switzerland, and in Torrejón el Rubio, Cáceres, Spain. In Zurich, the garden was located at the Irchel campus of the University of Zurich (47.3961 N, 8.5510 E, 508 m a.s.l.). In Torrejón el Rubio, the garden was situated at the Aprisco de Las Corchuelas research station (39.8133 N, 6.0003 W, 350 m a.s.l.). Zurich is characterized by a temperate climate, while the Spanish site is located in a dry, Mediterranean climate. The main climatic differences during the respective growing seasons (i.e., between April and August in Switzerland and between February and June in Spain) were precipitation (587 mm in Zurich, 229 mm in Cáceres) and daily average hours of sunshine (5.7 h in Zurich, 8.8 h in Cáceres). Temperatures during the growing season did not vary substantially between the two sites: averages of daily mean, minimum and maximum temperatures were 15.8, 10.9, and 21.1°C in Zurich versus 15.5, 9.7, and 21.4°C in Cáceres. All climatic data come from the respective national meteorological archives and are average values over the years 2000–2018.^1^

The experimental gardens were irrigated during the growing season with the aim of maintaining the above-mentioned differences in precipitation between the two sites while assuring survival of the crops during drought periods. In Spain, the automated irrigation system was configured for a dry threshold of soil moisture of 17% of field capacity, with a target of 25%. In Switzerland, the dry threshold was set at 50% of field capacity, with a target of 90% of field capacity. Whenever dry thresholds were reached (measured through PlantCare soil moisture sensors (PlantCare Ltd., Switzerland), irrigation was started and water added until reaching the target value.

Each experimental garden consisted of square plots of 0.25 m^2^ with a depth of 40 cm. Beneath 40 cm the plots were open, allowing unlimited root growth. The plots were embedded into larger beds: in Switzerland, there were 14 beds of 14 m × 1 m, each bed containing 28 plots. In Spain, beds were 20 m × 1 m and contained 40 plots. Inside a bed, plots were separated from each other by metal frames. Each plot was filled until 30 cm with standard, not enriched, agricultural soil coming from the local region. Therefore, soil structure and composition varied between sites and reflected the environmental histories of each site. In Spain, soil was composed of 78% sand, 20% silt, and 2% clay, and contained 0.05% nitrogen, 0.5% carbon, 253 mg total P/kg. In Switzerland, the soil consisted of 45% sand, 45% silt, and 10% clay, and contained 0.19% nitrogen, 3.39% carbon, and 332 mg total P/kg. Spanish and Swiss soils had a mean pH of 6.30 and 7.25, respectively (Stefan et al., 2021).

We fertilized half of the beds with nitrogen, phosphorus, and potassium at the concentration of 120 kg/ha N, 205 kg/ha P, and 120 kg/ha K. Fertilizers were applied three times, namely once just before sowing (50 kg/ha N, 85 kg/ha P, 50 kg/ha K), once when wheat was at the tillering stage (50 kg/ha N, 85 kg/ha P, 50 kg/ha K), and once when wheat was flowering (20 kg/ha N, 34 kg/ha P, 20 kg/ha K). The other half of the beds served as unfertilized controls. We randomly allocated individual beds to a fertilized or non-fertilized control treatment (Supplementary Figure 1).

### Crop Species

At each site, experimental communities were constructed with eight annual crop species. We used crop species belonging to four different phylogenetic groups with varying functional characteristics: *Triticum aestivum* (wheat, C3 grass) and *Avena sativa* (oat, C3 grass), *Lens culinaris* (lentil, legume) and *Lupinus angustifolius* (lupin, legume), *Linum usitatissimum* [flax, herb (superrosids)] and *Camelina sativa* [false flax, herb (superrosids)], and *Chenopodium quinoa* [quinoa, herb (superasterids)] and *Coriandrum sativum* [coriander, herb (superasterids)]. The crop cultivars that we chose were commercially available in Switzerland (the list of cultivars and suppliers can be found in Supplementary Table 1).

### Experimental Crop Communities

Experimental communities consisted of control plots with no crops, monocultures, 2- and 4-species mixtures. We planted every possible combination of 2-species mixtures with two species from different phylogenetic groups and every possible 4-species mixture with a species from each of the four different phylogenetic groups present. We replicated the experiment two times with the exact same species composition in each country. Within each country, we replicated the complete setup with and without fertilizer; plots were randomized within each fertilizer treatment, with each plot receiving the same amount of fertilization in the fertilized treatment. Each monoculture and mixture community consisted of one, two or four species planted in four rows. Each row only consisted of a single species. Two species mixtures were organized following a speciesA| speciesB| speciesA| speciesB pattern, where the described pattern refers to the row in which they were planted. The order of the species was chosen randomly. For 4-species mixtures, i.e., A| B| C| D, the order of the rows was also randomized. Density of sowing differed among species groups and was based on current cultivation practices: 160 seeds/m^2^ for legumes, 240 seeds/m^2^ for superasterids, 400 seeds/m^2^ for cereals, and 592 seeds/m^2^ for superrosids. Seeds were sown by hand in early February 2018 in Spain and early April 2018 in Switzerland. The plots were lightly tilled before sowing (at a depth of 5 cm). To sow, we dug four lines in the plots at 1–2 cm depth for all the species expect for the legumes, which were sown at 5 cm depth and for *Camelina*, which was sown at 0.5 cm. We then spread out the seeds in these lines and gently covered the surface. Germination and seedling establishment was controlled after two weeks; if germination was lower than 50% of the original seeds, additional seeds were resown to ensure that the plots would reach a sufficient plant density to allow plant interactions.

### Data Collection

#### Soil Samples

Soil samples were collected in each plot during flowering of the crops (early May in Spain, early June in Switzerland). We took three samples per plot to a depth of 25 cm, one between each of the four plant rows of a plot, which we then pooled. These soil samples were transported cooled to the lab and sieved with a 2 mm mesh size.

#### Soil Activity Measured as Soil Basal Respiration

With the sieved soil samples, we then measured water content, water holding capacity, and incubated 25 g equivalent dry soil at a normalized moisture level (60% WHC) for 12 h in airproof jars. The CO_2_ content in the headspace was measured with a LiCor LI820 once at the beginning of the incubation and once at the end. The difference between the two measures corresponded to the amount of CO_2_ that had been produced by soil microbial respiration (Curiel Yuste et al., 2007).

#### DNA Extraction and Amplification

Total nucleic acids were extracted from 250 mg of sieved soil using the DNeasy PowerSoil Kits (Qiagen, Hilden, Germany) following the supplier’s protocol. Concentration of extracted DNA was measured photospectrometrically with the QIAxpert System (Qiagen). Amplicon sequencing library construction was performed following a two-step PCR approach. The first step was performed using specific primers targeting the bacterial and archeal 16S ribosomal RNA gene (region V3–V4) and the fungal internal transcribed spacer region ITS2 using primer pairs 341F and 806R (Frey et al., 2016) and ITS3ngs and ITS4ngsUni (Tedersoo and Lindahl, 2016), respectively, including the sequencing primer sites of the Illumina adapters P5 (CTTTCCCTACACGACGCTCTTCCGATCT) and P7 (GGAGTTCAGACGTGTGCTCTTCCGATCT) required for the second step index PCR (Illumina Inc., San Diego, CA, United States). PCR amplification was performed in a volume of 25 μl containing 20 ng of template DNA, 1x GoTaq^®^ Flexi Buffer (Promega, Madison, WI, United States), 2.5 mM MgCl_2_ (Promega), 0.4 μM of each primer (Microsynth, Balgach, Switzerland), 0.2 mM dNTPs (Promega), 0.6 mg/ml BSA (VWR, Radnor, PA, United States) and 1.25 U GoTaq^®^ G2 Hot Start Polymerase (Promega). The PCR conditions consisted of an initial denaturation at 95°C for 5 min, 28 (16S rRNA gene) or 35 (ITS2 rrn) cycles of denaturation at 95°C for 40 s, annealing at 58°C for 40 s and elongation at 72°C for 1 min, followed by a final elongation at 72°C for 10 min. Each sample was amplified in triplicates and subsequently pooled. Pooled DNA samples were sent to the Functional Genomics Center Zurich (FGCZ, Zurich, Switzerland) for the indexing PCR. Index PCR products were purified, quantified, and pooled in equimolar ratios prior to sequencing on the Illumina MiSeq platform using the v3 chemistry (Illumina Inc.).

#### Soil Moisture Level

At the same time as the collection of soil samples, volumetric soil water content was measured from the soil surface to a depth of 6 cm using a ML3 ThetaProbe Soil Moisture Sensor (Delta-T, Cambridge). The measurements were taken in the center of each of the three in-between rows per plot and averaged per plot.

#### Grain Yield

Grain yield of each crop species was determined in each plot when grains reached maturity (duration of crop growth from sowing to harvest given in Table 1). As time of maturity slightly varied among the different crops, we harvested species by species.

**TABLE 1:** Sowing and harvesting dates, and crop growth duration in mean days after sowing from sowing until harvest for both countries and all eight species.

**Species**	**Sowing dates**		**Harvesting dates**		**Days after sowing**	
	**Switzerland**	**Spain**	**Switzerland**	**Spain**	**Switzerland**	**Spain**
*Avena sativa*	04.04.2018	02.02.2018	28.07.2018	27.06.2018	115	145
*Triticum aestivum*	04.04.2018	02.02.2018	28.07.2018	27.06.2018	115	145
*Lens culinaris*	04.04.2018	02.02.2018	12.08.2018	27.06.2018	130	145
*Lupinus angustifolius*	04.04.2018	02.02.2018	12.08.2018	17.06.2018	130	135
*Camelina sativa*	04.04.2018	02.02.2018	13.07.2018	02.07.2018	100	150
*Linum usitatissimum*	04.04.2018	02.02.2018	22.08.2018	12.07.2018	140	160
*Coriandrum sativum*	04.04.2018	02.02.2018	12.08.2018	27.06.2018	130	145
*Chenopodium quinoa*	04.04.2018	02.02.2018	01.09.2018	04.08.2018	150	183

### Data Analyses

Due to wild birds foraging on wheat seeds in Spain, and consequently, loss of data on wheat yield, we discarded the plots that were affected by the birds foraging. A total of 341 out of 384 plots remained, 181 in Switzerland and 160 in Spain.

#### Bioinformatics

Sequences were processed using a customized pipeline largely based on VSEARCH. The main steps included paired-end read merging using the *fastq_mergepairs* algorithm implemented in VSEARCH (Edgar and Flyvbjerg, 2015); primer trimming using Cutadapt (Martin, 2011) allowing for one mismatch; removing PhiX control reads using Bowtie2 (Langmead and Salzberg, 2012); quality filtering by maximum expected error of one using the *fastq_filter* function (Edgar and Flyvbjerg, 2015) implemented in VSEARCH; dereplicating sequences using the *derep_fulllength* function in VSEARCH; delineation of sequences into amplicon sequence variants (ASVs) using the *unoise3* algorithm (Edgar, 2016c) implemented as the *cluster_unoise* function in VSEARCH with an alpha of 2 and minsize of 4; removal of potentially chimeric sequences using the uchime2 algorithm (Edgar, 2016b) implemented as the *uchime3_denovo* function in VSEARCH; target verification using Metaxa2 (Bengtsson-Palme et al., 2015) and ITSx (Bengtsson-Palme et al., 2013) for the 16S rRNA gene and ITS2 sequences, respectively; mapping of the quality-filtered reads of each sample against the verified ASV sequences using the *usearch_global* function in VSEARCH; and taxonomic classification of each ASV sequence using the SINTAX classifier (Edgar, 2016a) implemented in VSEARCH with a bootstrap support of 0.8 against the SILVA database for the 16S rRNA sequences (Pruesse et al., 2007) and the UNITE database for the ITS2 sequences (Abarenkov et al., 2010). Non-fungal ITS2 sequences, as well as 16S rRNA sequences assigned to organelle structures (chloroplasts, mitochondria) were removed from the ASV table.

To remove effects of variability in sequencing read numbers, we performed 100-fold iterative subsampling of the ASV table using the function *rrarefy* from the *vegan* package in R (Oksanen et al., 2019; R Core Team, 2019), and subsequently computed the mean abundance for each ASV. Alpha diversity was assessed by calculating Shannon’s diversity (H) and Pielou’s evenness (J) indexes.

We used linear mixed models followed by type I analysis of variance to analyze the effects of the experimental treatment on fungal and bacterial ASV richness, H, and J, respectively. The analyses were performed for Spain and Switzerland separately. Fixed factors included fertilizing condition, crop species number (i.e., two or four crop species) nested in monoculture vs. mixtures, presence of cereal, presence of legume, presence of superrosid herb, presence of superasterid herb, as well as the interactions between them (except interactions between crop species number and monoculture vs. mixture and presence of functional group, respectively). Presences of the different functional groups were all binary factors (yes, no). Species composition and bed were set as random factors. Homogeneity of variance and normality of residues were assessed visually and with Shapiro-Wilk tests (Royston, 1982).

To analyze the responses of the microbial community composition to the experimental treatments, we first performed principal coordinates analysis (PCoA) using Bray-Curtis dissimilarity on relative ASV abundance (Gower, 1966). To that end, sparse ASVs (i.e., ASVs that appeared in one or two plots only) were removed, and relative abundance was log-transformed. We first performed this analysis taking both countries together, and in a second time, per country separately. Then, we used permutational multivariate analyses of variance (PERMANOVA; Anderson, 2001) with Bray-Curtis dissimilarity on the previously described composition matrices (i.e., separated per country), using the function *adonis* from the *vegan* package with 999 permutations (Oksanen et al., 2019). Experimental factors tested included fertilization, crop diversity treatments (monocultures vs. mixtures, crop species number, presence of the different functional groups and/or species), and their interactions. Homogeneity of variance was tested using permutational analysis of multivariate dispersion (PERMDISP; Anderson, 2006) implemented as the *betadisper* function in *vegan* with 999 permutations. Finally, we conducted a canonical analysis of principal coordinates (CAP; Anderson and Willis, 2003) for each country using the function *CAPdiscrim* from the *BiodiversityR* package (Kindt and Coe, 2005).

We analyzed species-specific and phylum-specific responses to the experimental factors with indicator species analyses using the *indicspecies* package (Cáceres and Legendre, 2009), calculating the point-biserial correlation coefficient, and correcting for multiple testing using the Benjamini-Hochberg method with the *p.adjust* function from the *stats* package (Benjamini and Hochberg, 1995).

We calculated total crop yield per plot (measured as the sum of grain mass of each species per plot), and the net biodiversity effect (NE) following the method from Hector and Loreau as the deviation from total expected yield in the mixture (Loreau and Hector, 2001). We then used the same linear mixed models as described above to analyse the response of total crop yield and NE to the experimental treatment variables, in Spain and in Switzerland, respectively.

#### Structural Equation Modeling

Considering the possibilities of direct and indirect effects of the different environmental and experimental variables (fertilizer, crop species richness) on soil moisture, soil microbes, soil respiration, and crop yield (measured as total grain mass per plot), we then applied structural equation modeling (SEM) to our data sets, separately for Switzerland and Spain. Because the effects of crop species number and crop community composition were correlated (for instance the 4-species mixtures automatically had one cereal, one legume, and two herbs) and thus difficult to disentangle in our study, we decided for simplicity of the SEM to only keep crop species number as a variable for crop diversity. The SEMs were built, run and evaluated with the *lavaan* package (Rosseel, 2012). We decided to fit the SEM to the Swiss and Spanish datasets separately because microbial composition in the two countries showed large differences. SEM allows to test complex *a priori* defined direct and indirect relationships in a unique framework and to assess the overall fit of the data to the model (Grace, 2006). For our *a priori* model, we considered environmental variables (fertilizer), crop species richness, soil moisture level in terms of volumetric water content, soil microbial alpha diversity measures (bacterial and fungal Shannon indexes), soil microbial beta diversity measures (coordinates from the three first axes of the principal coordinates analyses for fungi and bacteria, respectively), soil activity measured as CO_2_ flux, and total crop yield per plot. Our *a priori* model relating environmental and experimental factors, soil moisture, soil microbial diversity, soil activity and crop yield included the following hypotheses: (1) soil microbial diversity components (i.e., alpha diversity and community composition axes) are directly influenced by the environmental conditions (fertilizer), soil moisture, and the experimental treatment (crop species richness). (2) Soil activity can be affected directly or indirectly by crop species richness, soil moisture and fertilizer; the relationship can be indirect if effects on soil activity are mediated by effects on soil diversity measures. (3) Crop yield can also be directly or indirectly affected by crop species richness, soil moisture and fertilizer. The relationship can be indirect if effects on crop yield are mediated through changes in soil microbial diversity measures or soil activity. (4) All the soil microbial diversity measures can covary with each other.

To overcome scale differences among variables, we log-transformed crop yield before inclusion in the SEM. Path coefficients were estimated using maximum likelihood, and the model fits were tested with a chisquare goodness of fit test, a Bollen–Stine bootstrap test with 1000 bootstrap draws, a root mean square error of approximation (RMSEA) test, and the comparative fit index (CFI). A non-significant chisquare, Bollen–Stine and RMSEA test, as well as CFI values above 0.90, indicate a good fit of the model to the data (Kline, 2011).

Finally, we calculated the weighted average score of each ASV based on the previously mentioned PCoA decompositions, and subsequently investigated with linear models which bacterial and fungal genera were non-randomly distributed along the PCoA axes.

## Results

After sequencing, the fungal dataset contained 9,713,470 reads delineated into 7,856 ASVs. After removing all non-fungal ASVs, we obtained 6,161,288 reads assigned to 3,546 ASVs. The vast majority of the removed ASVs were assigned to plants despite having sieved the soils with 2 mm mesh size, which suggests low fungal biomass in our system. The bacterial dataset contained 6,394,402 reads delineated into 4,4136 ASVs. After removing all sequences associated with chloroplasts and mitochondria, we obtained 6,306,145 reads assigned to 43,857 ASVs.

### Soil Microbial Alpha Diversity

**FIGURE 1:**
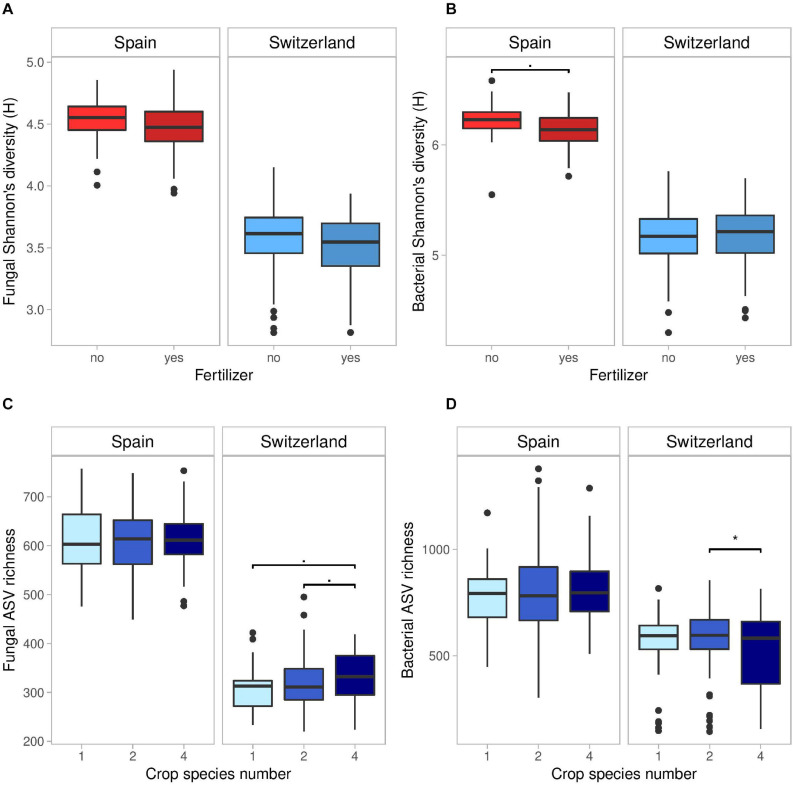
Effects of fertilizer on fungal **(A)** and bacterial **(B)** Shannon’s diversity index (H), and of crop species number on fungal **(C)**, and bacterial **(D)** ASV richness in Spain and Switzerland. Horizontal lines represent the median of the data, boxes represent the lower and upper quartiles (25 and 75%), with vertical lines extending from the hinge of the box to the smallest and largest values, no further than 1.5× the interquartile range. Data beyond the end of the whiskers are outlying and plotted individually. See Tables S2 and S3 for the results of the statistical analyses.

All diversity metrics were consistently lower in Switzerland compared to Spain (Figure 1): mean fungal ASV richness was 608 in Spain, against 324 in Switzerland (-47%), whereas fungal H was lower by 22% and fungal J by 13%. Similarly, mean bacterial ASV richness was lower by 30% (i.e., 803 vs. 561 ASVs in Spain vs. Switzerland); bacterial H was 16% lower and bacterial J 11% lower in Switzerland compared to Spain.

In Spain, bacterial H was marginally lower (2%) in fertilized compared to unfertilized plots (Supplementary Table 2 and Figure 1B). Furthermore, the presence of a superasterid herb marginally increased bacterial ASV richness (+2.5%) and decreased bacterial J (−1%).

In Switzerland, fertilization decreased bacterial ASV richness (−16% in fertilized compared to unfertilized plots) but increased bacterial evenness (+4%) (Supplementary Table 3). Fungal ASV richness marginally increased with crop species number (Figure 1C; +7% in 4-species mixtures compared to monocultures and +4% compared to 2-species mixtures), while bacterial ASV richness was higher in 2-species mixtures compared to 4-species mixtures (Figure 1D). Finally, the presence of a legume marginally decreased fungal H (−1%) and J (−1%) in comparison to plots without legumes, while cereal presence marginally decreased bacterial J (−2%).

### Soil Microbial Community Composition

Fungal and bacterial community composition differed strongly between countries (Supplementary Figure 2), with the country accounting for 66 and 52% of the variance in fungal and bacterial communities, respectively (Supplementary Table 4). All other factors explained less than 1% of the remaining variance. In Spain, fungal communities were characterized by higher relative abundance of *Ascomycota, Mortierellomycota*, and *Mucoromycota*, while the fungal communities in the Swiss soils had relatively more *Basidiomycota, Chytridiomycota*, and *Rozellomycota* (Supplementary Figures 3A, 4A). Notable changes in bacterial communities between the two countries include higher relative abundance of *Planctomycetes, Actinobacteria, Acidobacteria*, and *Nitrospirae* in Spain, while in Switzerland bacterial communities showed more *Proteobacteria, Chloroflexi, Bacteroidetes*, and *Cyanobacteria* (Supplementary Figures 3B, 4B).

In Spain, the community composition of both fungi and bacteria was significantly affected by fertilization, presence of cereal, as well as their interaction (Table 2 and Supplementary Figures 5, 6). The interaction between fertilizer and the presence of legume also had an effect on fungal communities in Spain. Fertilized plots had fungal communities with relatively more *Chytridiomycota*, while *Mortierellmycota, Mucoromycota*, and *Kickxellomycota* were positively linked to unfertilized plots (Supplementary Figure 7A). For bacterial communities, we observed a relative increase of *Proteobacteria, Actinobacteria, Nitrospirae, Firmicutes*, and *Patescibacteria* in fertilized plots, while *Planctomycetes, Acidobacteria*, and *Chloroflexi* were more abundant in unfertilized plots. *Deinococcus-Thermus* and *Fibrobacteres* increased in relative abundance in the presence of cereals, while *Nitrospirae* was associated with the absence of cereals (Supplementary Figure 9A).

**TABLE 2:** Results of the permutational analysis of variance, showing *R*^2^ and significance of the considered factors for the community composition of fungi and bacteria in Spain and Switzerland.

	**Fungi community composition**				**Bacteria community composition**			
	**Spain**		**Switzerland**		**Spain**		**Switzerland**	
	**R^2^**	**p-value**	**R^2^**	**p-value**	**R^2^**	**p-value**	**R^2^**	**p-value**
Fertilizer	**0.0471**	0.0010***	**0.0337**	0.0010***	**0.0427**	0.0010***	**0.0425**	0.0010***
Monoculture vs mixture	0.0054	0.8470	0.0062	0.2670	0.0070	0.2980	0.0055	0.3920
Crop species number (2 vs 4)	0.0068	0.3820	**0.0132**	0.0020**	0.0072	0.2420	**0.0104**	0.0040**
Cereal	**0.0156**	0.0010***	**0.0178**	0.0010***	**0.0093**	0.0380*	**0.0130**	0.0020**
Legume	0.0083	0.1130	**0.0165**	0.0020**	0.0079	0.1510	**0.0124**	0.0010***
Superasterid herb	0.0087	0.0640	**0.0093**	0.0320*	0.0069	0.3050	0.0077	0.0660
Fertilizer x mono vs mixture	0.0065	0.4630	0.0051	0.4970	0.0059	0.7190	0.0077	0.0590
Fertilizer x species number	0.0067	0.4210	0.0052	0.4820	0.0060	0.6740	0.0048	0.6960
Fertilizer x cereal	**0.0100**	0.0290*	0.0059	0.3150	**0.0101**	0.0240*	0.0055	0.4010
Fertilizer x legume	**0.0099**	0.0240*	0.0038	0.8580	0.0081	0.1270	**0.0079**	0.0430*
Fertilizer x superasterid herb	0.0054	0.8190	0.0055	0.4070	0.0062	0.5980	0.0069	0.1200

*Number of observations are 181 in Switzerland and 160 in Spain.*

*p*-Values are significant at α = 0.05.

*p < 0.05.

**p < 0.01.

***p < 0.001.

*Bold values indicate significant responses (*p*-values < 0.05).*

In Switzerland, fungal and bacterial communities were affected by fertilization, crop species number, presence of cereal and presence of legume (Table 2 and Supplementary Figures 5, 6). Fungal communities were also affected by the presence of superasterid herbs. The interaction of fertilization and presence of legume had an effect on bacterial communities. *Mucoromycota* were more abundant in fertilized plots, while *Ascomycota* were more present in unfertilized plots (Supplementary Figure 7B). For bacterial communities, we noticed an increase in *Proteobacteria, Patescibacteria, Hydrogenedentes, Bacteroidetes*, and *Cyanobacteria* in fertilized plots, while *Planctomycetes, Actinobacteria*, and *Firmicutes* were more abundant in unfertilized plots. Furthermore, there was an increase in the relative abundance of *Fibrobacteres, Verrucomicrobia*, and *Armatimonadetes* in the presence of cereals, while plots with no cereal had more *Hydrogenedentes* and *Latescibacteria* (Supplementary Figure 9B).

### Total Crop Yield and Net Biodiversity Effect (NE)

**FIGURE 2:**
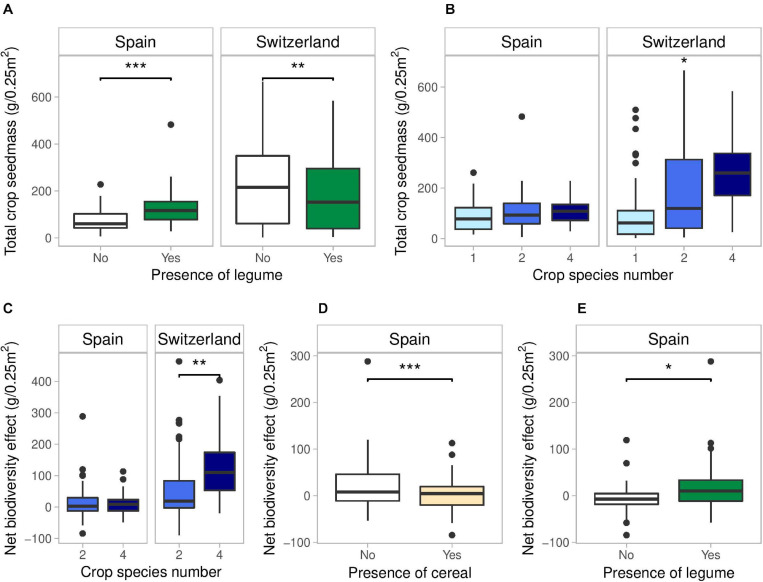
Effects of legume presence and crop species number on total crop yield **(A,B)**, and effects of crop species number, presence of cereal and legume on Net biodiversity Effects **(C–E)** in Spain and Switzerland. Horizontal lines represent the median of the data, boxes represent the lower and upper quartiles (25 and 75%), with vertical lines extending from the hinge of the box to the smallest and largest values, no further than 1.5 × the interquartile range. Data beyond the end of the whiskers are outlying and plotted individually. See Tables S5, S6, S7 and S8 for the results of the statistical analyses. *p < 0.05, **p < 0.01, and ***p < 0.001.

Total crop yield had an average of 416 g/m^2^ in Spain and 784 g/m^2^ in Switzerland. In Spain, total crop yield was only increased by the presence of a legume (+72%) (Figure 2A and Supplementary Table 5). In Switzerland, total crop yield significant increased with crop species number (Figure 2B and Supplementary Table 6): two-species and four-species mixtures showed an increase in average yield of 43 and 102% compared to monocultures, while 4-species mixture showed an increase in average yield of 41% compared to 2-species mixtures. The presence of a cereal and a superasterid herb increased total yield in Switzerland (+102%, +96%), while the presence of a legume decreased total yield (-20%) (Figure 2A).

Net biodiversity effect was positive in both countries, which means that the mixtures consistently overyielded. However, NE was much higher in Switzerland compared to Spain (+680%) (Figure 2C). In Spain, NE was significantly reduced by the presence of a cereal (−108%) and increased in the presence of a legume (+219%) (Figures 2D,E and Supplementary Table 7). In Switzerland, NE was significantly higher in 4-species mixtures compared to 2-species mixtures (+150%) (Figure 2C and Supplementary Table 8).

### Structural Equation Modeling

Our data showed overall good fit to our *a priori* SEM in Spain and Switzerland: chisquare = 3.828 (Spain) and 4.939 (Switzerland); P(chisquare) = 0.281 (Spain) and 0.173 (Switzerland); P(Bollen–Stine Bootstrap) = 0.459 (Spain) and 0.464 (Switzerland); RMSEA = 0.046 (Spain) and 0.064 (Switzerland); P(RMSEA) = 0.415 (Spain) and 0.321 (Switzerland); CFI = 0.999 (Spain) and 0.997 (Switzerland).

**FIGURE 3:**
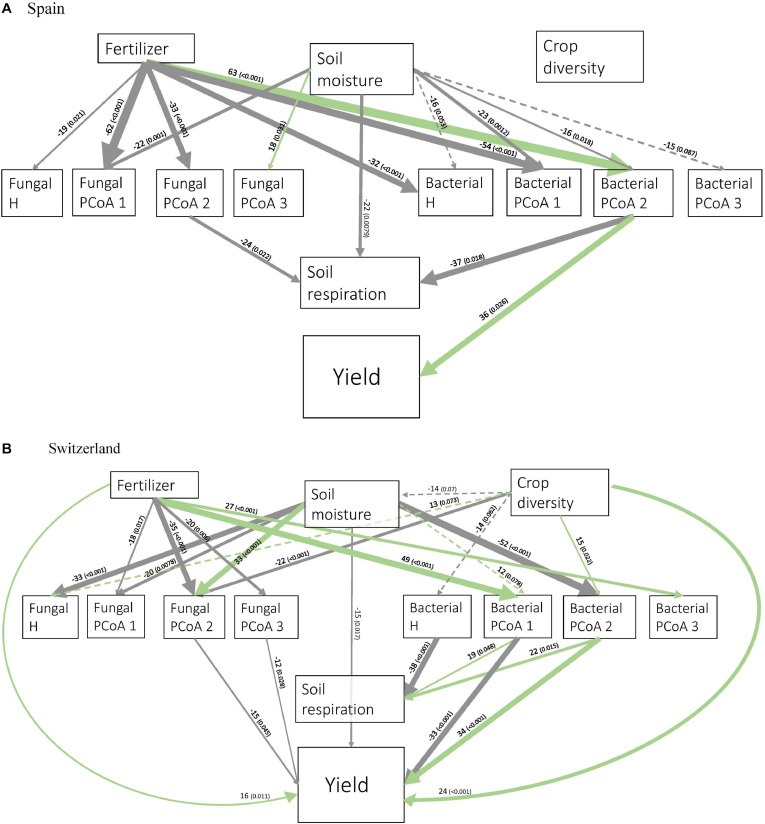
Structural Equation Modeling showing the relationships between crop diversity, fertilizer, soil moisture, soil activity, and bacterial and fungal diversity measures in Spain **(A)** and Switzerland **(B)**. H: Shannon’s diversity. Only significant (solid line) and marginally significant (dashed line) relationships are shown. Width of arrows are proportional to the strength of the standardized path coefficients indicated by the numbers above the arrows. The numbers in brackets indicate associated *p*-values. Colors of the arrows show positive (green) and negative (gray) effects. Residual correlations are not shown.

In Spain, soil diversity measures were mostly influenced by fertilizer and soil moisture (Figure 3A). Soil microbial activity was directly influenced by soil moisture, fungal PCoA 2 and bacterial PCoA 2. Crop yield was only linked to bacterial PCoA axis 2. Bacterial PCoA axis 2 was associated with a response of *Bacilli, Actinobacteria* and *Acidobacteria*, among others (Supplementary Figure 11), which therefore correlated with an increase in crop yield in our study. Fertilization had an indirect positive effect on crop yield via changes in bacterial PCoA axis 2.

In Switzerland, fertilizer and soil moisture also significantly influenced almost all the soil diversity measures (Figure 3B). Moreover, crop diversity had a direct effect on soil moisture, bacterial and fungal H, fungal PCoA axis 2, and bacterial PCoA axis 2. Soil microbial activity was negatively affected by bacterial H, and positively by the first and second axes of bacterial PCoA. Crop yield was directly affected by some soil diversity measures (fungal PCoA axes 1 and 2, bacterial PCoA axes 1 and 2), and also by crop diversity, fertilizer and soil moisture.

Crop diversity indirectly increased soil activity in Switzerland through changes in bacterial H and the second axis of bacterial PCoA.

**FIGURE 4:**
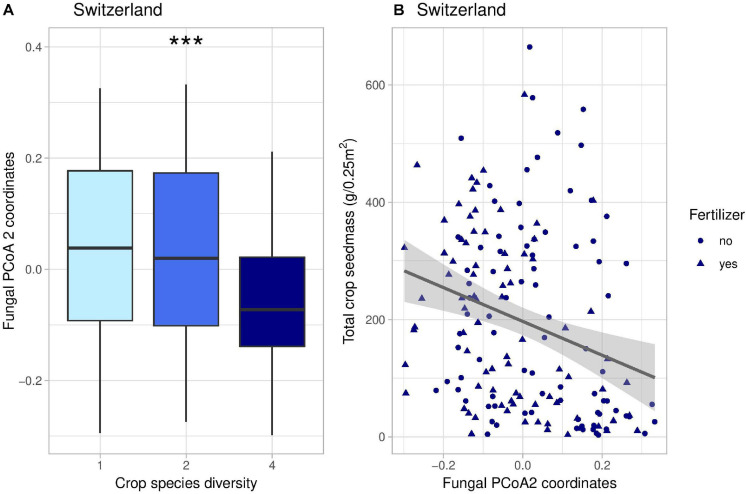
Effects of crop species number on the coordinates of the second axis of fungal PCoA decomposition **(A)** and correlation between these coordinates and total crop yield **(B)** in Switzerland. In panel **(B)**, the line represents the linear regression (coefficient: −288, *p*-value = 0.00037). *** indicates the significance level of the effects of crop species number on fungal PCoA 2 (*p*-value < 0.001).

**FIGURE 5:**
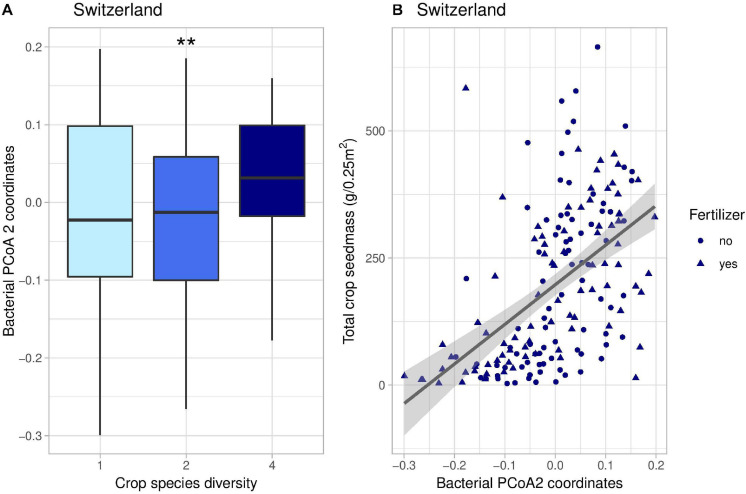
Effects of crop species number on the coordinates of the second axis of bacterial PCoA decomposition **(A)** and correlation between these coordinates and total crop yield **(B)** in Switzerland. In panel **(B)**, the line represents the linear regression (coefficient: 779, *p*-value < 0.001). **indicates the significance level of the effects of crop species number on bacterial PCoA 2 (*p*-value < 0.01).

**FIGURE 6:**
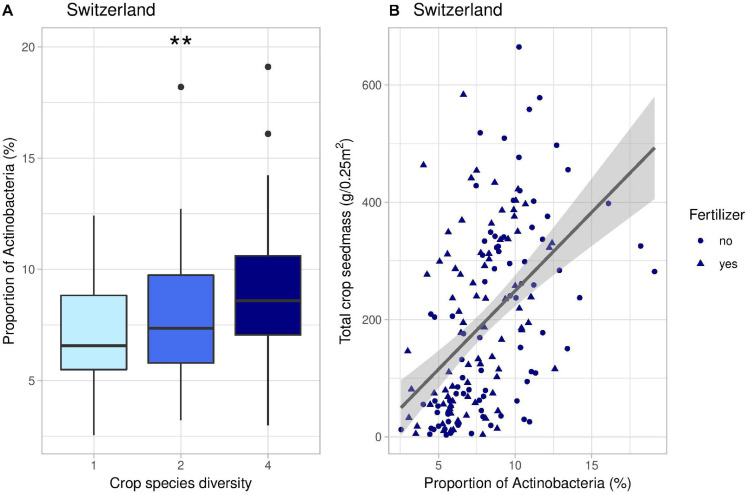
Effects of crop species number on the proportion of Actinobacteria **(A)** and correlation between the proportion of Actinobacteria and total crop yield **(B)** in Switzerland. In panel **(B)**, the line represents the linear regression (coefficient: 26.8, *p*-value < 0.001). **indicates the significance level of the effects of crop species number on bacterial PCoA 2 (*p*-value < 0.01).

Crop diversity indirectly affected crop yield in Switzerland through five different pathways. Firstly, there were two indirect positive effects through the second axis of fungal PCoA (Figure 4); fungi genera associated with negative coordinates along the PCoA axis 2 included for instance *Kondoa, Serendipita, Mucor*, or *Leucospiridium*, among others (Supplementary Figure 10). The presence of these fungal genera is therefore linked to an increase in crop yield. Secondly, there were two positive effects of crop diversity on crop yield mediated by the second axis of bacterial PCoA (Figure 5). Bacterial PCoA axis 2 is related to a response of *Nitriliruptoria, Chloroflexia, Acidimicrobia*, and *Actinobacteria*, among others (Supplementary Figure 12B); these bacteria thus form another potential group of yield promoting soil microbes in our study (Figures 5, 6). Finally, crop diversity indirectly increased crop yield through the first axis of bacterial PCoA. Bacterial genera associated with negative coordinates along the PCoA axis 1 include *Nitriliruptoria, Actinobacteria, Acidimicrobia, Chloroflexia*, and *Bacilli*, among others (Supplementary Figure 12A), i.e., a third group of yield promoting soil microbes in our study (Figure 6). Fertilization had an indirect positive effect on crop yield, via changes in fungal PCoA axes 2 and 3, but also an indirect negative effect on crop yield, mediated by changes in bacterial PCoA axis 1.

## Discussion

Our study shows that fertilization, as well as—in some cases—crop diversity and composition, can affect soil microbial community composition, and that these compositional changes can go along with changes in crop yield. In particular, the data suggests that in Switzerland, the positive effects of increasing crop diversity on crop yield are partially mediated by changes in microbial composition, notably through a response of plant-growth promoting bacteria. However, as expected, changes in soil microbes do not fully explain yield variations and thus, other processes must play an important role in increasing crop yield in mixtures. Furthermore, the effects of crop diversity on microbial communities remained small compared to the effects of fertilization. In Spain, where soil resources were limited and both crop and soil communities experienced more stressful environmental conditions than in Switzerland, we did not observe any significant responses of soil microbes to crop diversity. We suggest that this context-dependency of crop diversity effects on soil microbes and ecosystem functioning might be explained by differences in abiotic factors; however, further research in various environmental conditions is needed to better understand the reasons behind this context-dependency.

### Crop Species Number Effects on Microbes

Our first hypothesis was that an increase in crop diversity would lead to an increase in microbial alpha diversity as well as changes in soil microbial community composition. We did observe an increase in fungal ASV richness in response to crop diversity, but this effect was only marginally present in Switzerland. This is consistent with results from studies in natural environments, where plant species richness positively correlated with fungal richness (Yang et al., 2017). There was no effect of crop diversity on any of the bacterial alpha diversity measures; however, in both countries, we observed changes in bacterial and fungal beta diversity in response to crop diversity and crop composition (Table 2). In Switzerland notably, we observed a shift in soil fungal and bacterial composition between 2-species mixtures and 4-species mixtures. This is in agreement with previous research demonstrating that plant diversity did not correlate with microbial alpha diversity, but it did with beta diversity (Grüter et al., 2006; Prober et al., 2015). Moreover, crop composition also affected microbial communities: for instance, the presence of a cereal induced changes in fungal and bacterial soil communities in both countries, with a notable increase in abundance of *Fibrobacteres, Armatimonadetes*, and *Verrucomicrobia.* The two latter groups have commonly been found in the rhizosphere of wheat or oat, even though little is known about their functions (Rascovan et al., 2016; Dai et al., 2020; Lupwayi et al., 2020). The response of *Fibrobacteres*—which are mostly involved in the degradation of plant-based cellulose (Gupta, 2004)—to the presence of a cereal has been scarcely mentioned in previous research (Mahoney et al., 2017). These changes in microbial composition may be due to above-ground processes linked to crop diversity and composition, such as variations in microclimate associated with plant canopy cover (Delgado-Baquerizo et al., 2018), or below-ground processes, such as increased diversity of root exudates or plant signaling molecules (Eisenhauer et al., 2017); however, more research is needed to investigate the mechanisms behind these specific responses.

Secondly, we hypothesized that crop diversity would enhance soil activity; this was indeed the case in Switzerland, where crop diversity increased soil respiration via changes in bacterial richness and composition (Figure 3B). This is consistent with grassland studies demonstrating a positive link between plant diversity and soil microbial activity (Lange et al., 2015; Steinauer et al., 2015), possibly due to increases in root inputs, changes in root exudates (Bais et al., 2006), or a reduction in evapotranspiration from the topsoil due to denser vegetation in diverse plant communities (Lange et al., 2014).

### Soil Microbes Partially Explain the Positive Effects of Intercropping on Yield in Switzerland

Results from our SEM in Switzerland suggest that the changes in soil microbial communities induced by crop diversity further affected crop yield (Figure 3B). We had previously shown that there was a positive effect of crop species richness on crop yield in Switzerland, demonstrating a positive diversity–productivity relationship (Figure 2B; Chen et al., 2020; Engbersen et al., 2020). Here, we provide evidence that these positive effects of intercropping on crop yield can partially be explained by changes in microbial communities. Indeed, increasing crop diversity was linked to an increase in yield through five indirect pathways mediated by changes in fungal or bacterial composition (Figure 3B). We noticed that those changes in microbial composition associated with increases in crop yield were characterized by a response of potentially beneficial bacteria such as plant growth-promoting rhizobacteria (PGPRs). For instance, this was the case of *Actinobacteria* (Figure 6 and Supplementary Figure 11, 12), which are known to be symbionts of plants as well as saprophytes (Bhattacharyya and Jha, 2012). They also play a role as biocontrol agents against a range of pathogenic fungi and promote plant growth (Merzaeva and Shirokikh, 2006; Franco-Correa et al., 2010). Some examples include *Glycomyces, Agromyces*, or *Nocardioides*, which are genera of PGPRs (Qin et al., 2011; Hamedi and Mohammadipanah, 2014), and *Streptomyces*, known to associate with wheat and possessing anti-fungal properties (Conn and Franco, 2004). Other potential PGPRs that were found to positively correlate with crop yield in our study include *Pseudomonas, Burkholderia, Bacillus*, and *Massilia* (Supplementary Figure 11, 12). *Pseudomonas* has been shown to promote plant growth as well as inhibit pathogenic fungi (Gray and Smith, 2005; Melo et al., 2016). *Burkholderia* and *Bacillus* can be involved in phosphate solubilization (Dimkpa et al., 2009; Zhao et al., 2014) and symbiotic associations with wheat (Shaharoona et al., 2007; Moreno-Lora et al., 2019). Finally, *Massilia* has been shown to correlate with an increase in plant biomass for alfalfa and soybean (Xiao et al., 2017). Some bacteria involved in nitrogen cycling also positively correlated with the PCoA coordinates leading to an increase in crop yield in Switzerland, such as *Nitrosospira*—ammonia-oxidizing genus (Fierer et al., 2007), and *Nitrolancea*—nitrite-oxidizing genus (Daims et al., 2016).

When looking at fungi, our results showed that in Switzerland crop yield was positively associated with fungal genera including *Claroideoglomus, Myrmecridium, Serendipita, Mucor*, as well as several yeasts (e.g., *Torula, Kondoa, Leucosporidium*) (Supplementary Figure 10). *Claroideoglomus* belongs to the *Glomerales* order, which are biotrophic mutualists and can establish arbuscular mycorrhizal networks with wheat (Dai et al., 2014). *Myrmecridium* and *Mucor* are saprophytes, involved in decomposition of organic matter and nutrient cycling (Schlatter et al., 2018), while *Serendipita* are plant growth promoting fungi which have been shown to have beneficial effects on many plants, including wheat (Singhal et al., 2017). Finally, yeasts have also been suggested as potential bio-agents and plant growth promoters (Mukherjee et al., 2020); furthermore, they are a nutrient source for some bacteria and contribute to essential soil ecological processes such as mineralization of organic matter (Botha, 2011). Among the fungal genera associated with positive PCoA coordinates, i.e., lower crop yield, we found a few plant pathogens, such as *Protomyces*, causing stem gall disease in coriander (Khan and Parveen, 2018), and *Pyrenochaeta*, a parasite for plant roots (Aragona et al., 2014). Our study thus suggests that in Switzerland, increasing crop diversity leads to changes in microbial communities that enhance the presence of beneficial microbes and reduce pathogen loads, which may promote plant growth and contribute to the observed increase in crop yield.

### The Importance of Microbial Communities Is Relative and Context-Dependent

While changes in soil microbial composition explained part of the positive effects of crop diversity on crop yield, there were also direct effects of intercropping on yield (i.e., not mediated by microbes) in Switzerland. This demonstrates that other mechanisms must play a role in increasing crop productivity in diverse mixtures. These processes could include a complementary use of resources, such as nutrient or light partitioning (Jesch et al., 2018; Engbersen et al., 2020), trait differentiation (Cadotte, 2017), or changes in crop–weed interactions (Stefan et al., 2020).

Moreover, our study highlights the context-dependency and relative importance of crop diversity effects on soil microbes. Indeed, our results suggest that abiotic factors such as fertilization or soil moisture were more important drivers of soil microbial communities than crop diversity. This is consistent with previous research indicating that soil microbial community composition mainly depends on soil moisture, temperature, and organic matter contents, and that these factors are often more important than crop diversification (Ren et al., 2018; Guo et al., 2020). In our case, in Switzerland, the effects of soil moisture and fertilization on microbial composition were indeed up to three times stronger than the effect of crop diversity (Figure 2B). Interestingly, fertilization in Switzerland seemed to have conflicting indirect effects on yield: on the one hand, it promoted fungal communities that were beneficial for crop yield, such as *Mucoromycota* (Supplementary Figure 7B), but on the other hand, it also favored bacteria that were not positively associated with crop yield, such as *Proteobacteria*. Bacterial phyla responding to mineral fertilization included *Proteobacteria, Hydrogenedentes, Bacteroidetes*, and *Cyanobacteria* (Supplementary Figure 8B). This is consistent with previous studies demonstrating that the relative abundance of *Proteobacteria* and *Bacteroidetes* generally increase under high-N conditions (Fierer et al., 2012). *Bacteroidetes* and many proteobacterial groups have been identified as copiotrophic taxa, which tend to thrive in resource-rich environments (Fierer et al., 2007; Ramirez et al., 2010). On the contrary, *Actinobacteria*, which include many PGPRs, was significantly more abundant in unfertilized plots (Supplementary Figure 8B). Therefore, we might hypothesize that high-N conditions would favor the growth of *Proteobacteria* at the expense of *Actinobacteria* (Lupwayi et al., 2018), thereby limiting the abundance of plant beneficial bacteria.

In Spain, soil microbes responded to soil moisture and fertilization but did not respond to crop diversity. We propose several reasons for this lack of crop–microbe relationship in Spain, while acknowledging the speculative character of the following suggestions due to the limitations of having one unique site per country. First, soil texture in Spain was dominated by sand (78%) with only a small proportion of clay (2%), unlike Switzerland where sand and silt were in equal proportions (45%) with a higher clay content (10%). Higher clay and silt content usually protect and favor microbial biomass (Bach et al., 2010) through larger aggregates (Wick et al., 2009), greater water holding capacity (Voroney, 2007), and increased nutrient retention (Knops and Tilman, 2000). This is supported by the fact that microbial DNA concentration was indeed much lower in Spain (Supplementary Figure 13), which indicates that the soil microbial communities were poorer in Spain in terms of biomass, and consequently perhaps more resistant to change (Landesman and Dighton, 2010). Secondly, the climate in Spain is more arid than in Switzerland, which led to lower soil moisture and increased water stress for plant and soil communities. Also, soil nutrient content was lower in Spain. Spanish soil communities thus experienced greater abiotic stress than in Switzerland, which may be why they primarily responded to soil moisture or fertilization—factors that can directly alleviate their stress—rather than crop diversity. Furthermore, there was almost no link between microbial communities and crop yield in Spain; in particular, fungal communities did not have any effect on crop yield, while only one axis of the bacterial composition positively affected yield in response to fertilization (Figure 3A). This decoupling between yield and soil microbes suggests that crop productivity in Spain might be limited by other, more impactful factors that could overshadow any potential effects of microbes, such as nutrient or water availability. This is consistent with a recent study by Adomako et al. (2020), in which they found that the positive effects of soil microbes on plant biomass were stronger under high nutrient and water availability compared to low resource conditions. Indeed, in conditions of nutrient stress, microbial communities may compete for the limited available nutrients (Ho and Chambers, 2019). If the amount of nutrients is insufficient, it is possible that microbes cannot exchange nutrients for carbon with the plants anymore, which can lead to negative plant–soil feedbacks (Petermann et al., 2008; Zandt et al., 2019). In the other way around, nutrient or water limitation, by restricting plant growth—as it was the case in Spain (Figure 2A)—might also reduce the amount of carbon provided by the plants to fuel the microbial communities (Harrison and Bardgett, 2010). This reduction in plant–soil feedbacks raises important questions on the universality of the role of soil microbes for plant growth, and calls for more experiments in various soil and climatic conditions to confirm and improve our understanding of crop–soil microbes relationships in agricultural systems.

## Conclusion

Our study shows that in some cases, crop diversity has the potential to influence soil microbial community composition and that these changes partially explain the positive effects of intercropping on yield. However, these effects are relative and less important than changes in abiotic factors, such as the addition of mineral fertilizer. Furthermore, these processes are context-dependent: in Spain, soil microbial communities do not respond to crop diversity, and their effect on crop yield is weaker. We hypothesize that these contrasting responses could be explained by differences in abiotic conditions and resources. However, further investigation is required to test this context-dependency hypothesis. Considered as a whole, this research suggests that soil microbial communities can play a beneficial role in intercropped systems. Yet this positive result is not universal; on the contrary, it reflects on the relative importance of microbial communities compared to abiotic factors for increasing crop productivity, and highlights the context-dependency of crop–microbe relationships.

## Data Availability Statement

The datasets presented in this study can be found in online repositories. The names of the repository/repositories and accession number(s) can be found below: https://doi.org/10.5281/zenodo.4479277.

## Author Contributions

LS and CS conceived the study with input from MH, JS, and NE. LS, NE, and CS collected the data. LS assembled and analyzed the data with the help of CS and MH. LS, MH, and CS wrote the manuscript. All authors discussed the data analyses and results.

## Conflict of Interest

The authors declare that the research was conducted in the absence of any commercial or financial relationships that could be construed as a potential conflict of interest.

## Footnotes

1 www.meteoschweiz.admin.ch, www.datosclima.es
