# Supplementary material for: Positive Effects of Crop Diversity on Productivity Driven by Changes in Soil Microbial Composition
Source: Front Microbiol. 2021 Apr 15;12:660749. doi: 10.3389/fmicb.2021.660749 (PMC8081861; doi:10.3389/fmicb.2021.660749)
Supplement: Supplementary file 1 [file Data_Sheet_1.PDF]

Supporting Information

Table S 1. List of crop species cultivars and their suppliers in Switzerland.

Species	Cultivar	Supplier
Avena sativa	Canyon	Sativa Rheinau
Triticum aestivum	Fiorina	DSP, Delley
Coriandrum sativum	Indian	Zollinger Samen, Les Evouettes
Chenopodium quinoa	n.a.	Artha Samen, Münsingen
Lupinus angustifolius	Boregine	Aspenhof, Wilchingen
Lens culinaris	Anicia	Agroscope, Reckenholz
Camelina sativa	n.a.	Zollinger Samen, Les Evouettes
Linum usitatissimum	Lirina	Sativa Rheinau

Figure S1. Schematic representation of the mesocosm experiments. The fertilizer and crop diversity treatments were fully randomized within the two countries.

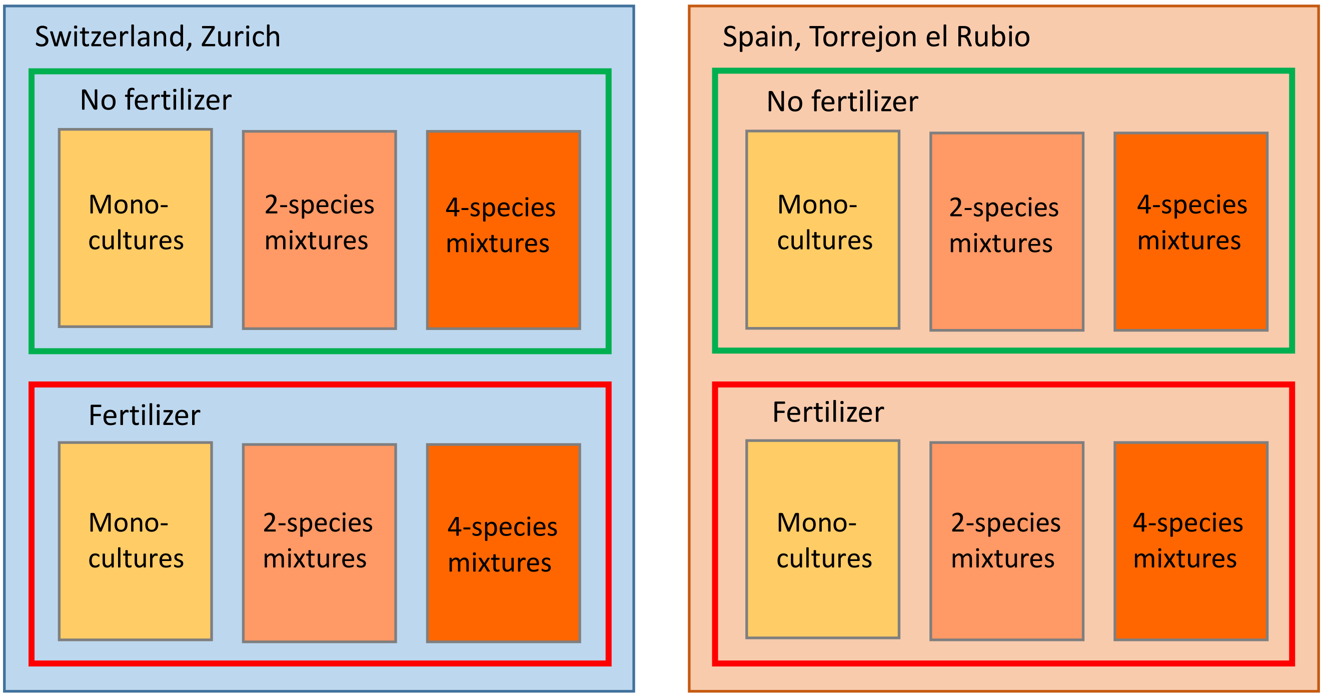

Table S2. Results of mixed effects ANOVA testing environmental factors, crop diversity and functional group presence on fungal and bacterial Shannon's index (H), ASV richness, and Pielou's evenness index (J) in Spain. Numbers between brackets indicate the p-value.
P-values are significant at α = 0.05 and highlighted in bold.

	F-value H fungi	*F-value ASV richness fungi*	*F-value J fungi*	*F-value H bacteria*	*F-value ASV richness bacteria*	*F-value J bacteria*
Fertilizer	3.0221 (0.11)	3.0887 (0.11)	1.0822 (0.35)	4.4977 (0.057)	2.5942 (0.13)	1.3684 (0.26)
Mono_mix	0.0258 (0.87)	0.222 (0.64)	0.1003 (0.75)	0.0219 (0.88)	0.3531 (0.55)	0.0433 (0.84)
Crop species number	0.3273 (0.57)	0.0368 (0.85)	0.422 (0.52)	1.0894 (0.30)	0.0076 (0.93)	0.0987 (0.75)
Cereal	0.2246 (0.64)	0.2071 (0.65)	0.4464 (0.51)	0.8391 (0.36)	1.5679 (0.21)	0.6699 (0.41)
Legume	2.1066 (0.15)	0.6455 (0.42)	1.7165 (0.19)	0.927 (0.34)	0.4778 (0.49)	0.0294 (0.86)
Superasterid herb	0.0829 (0.77)	0.3017 (0.58)	0.0066 (0.94)	2.5946 (0.11)	3.7348 (0.056)	**5.892 (0.017)**
Fertilizer x mono_mix	0.0062 (0.94)	1.2457 (0.27)	0.182 (0.67)	0.0012 (0.97)	0.0765 (0.78)	1.0108 (0.32)
Fertilizer x crop sp.number	0.0634 (0.80)	0.2356 (0.63)	0.2357 (0.63)	0.2191 (0.64)	0.0132 (0.91)	1.0014 (0.32)

Table S3. Results of mixed effects ANOVA testing environmental factors, crop diversity and functional group presence on fungal and bacterial Shannon's index (H), ASV richness, and Pielou's evenness index (J) in Switzerland. Numbers between brackets indicate the p-value.
P-values are significant at α = 0.05 and highlighted in bold.

	F-value H fungi	*F-value ASV richness fungi*	*F-value J fungi*	*F-value H bacteria*	*F-value ASV richness bacteria*	*F-value J bacteria*
Fertilizer	2.3161 (0.13)	2.8237 (0.12)	0.0541 (0.82)	0.0027 (0.96)	**7.0037 (0.022)**	**24.8374 (0.00060)**
Mono_mix	1.4202 (0.24)	2.0425 (0.15)	1.2216 (0.27)	1.8163 (0.19)	0.0377 (0.85)	2.3764 (0.13)
Crop species number	2.1966 (0.15)	2.964 (0.087)	1.9326 (0.17)	2.8809 (0.097)	**4.3801 (0.038)**	0.0098 (0.92)
Cereal	0.3229 (0.57)	0.7977 (0.37)	1.9207 (0.17)	2.5043 (0.12)	0.2807 (0.60)	**5.547 (0.020)**
Legume	**4.4919 (0.04)**	3.7824 (0.054)	**5.5246 (0.02)**	2.1157 (0.15)	1.2589 (0.26)	2.1281 (0.15)
Superasterid herb	0.4028 (0.53)	0.3254 (0.57)	1.8028 (0.18)	0.0072 (0.93)	0.575 (0.45)	1.9106 (0.17)
Fertilizer x mono_mix	0.5653 (0.45)	1.0359 (0.31)	0.7852 (0.38)	**4.3963 (0.038)**	0.5868 (0.44)	0.4237 (0.52)
Fertilizer x crop sp.number	0.1705 (0.68)	0.9682 (0.33)	0.1114 (0.74)	1.1454 (0.29)	0.9522 (0.33)	0.1176 (0.73)

Figure S1. Principal Coordinates Analysis plot of fungal (left) and bacterial (right) communities. Spanish samples are represented in red, while Swiss samples are represented in blue.

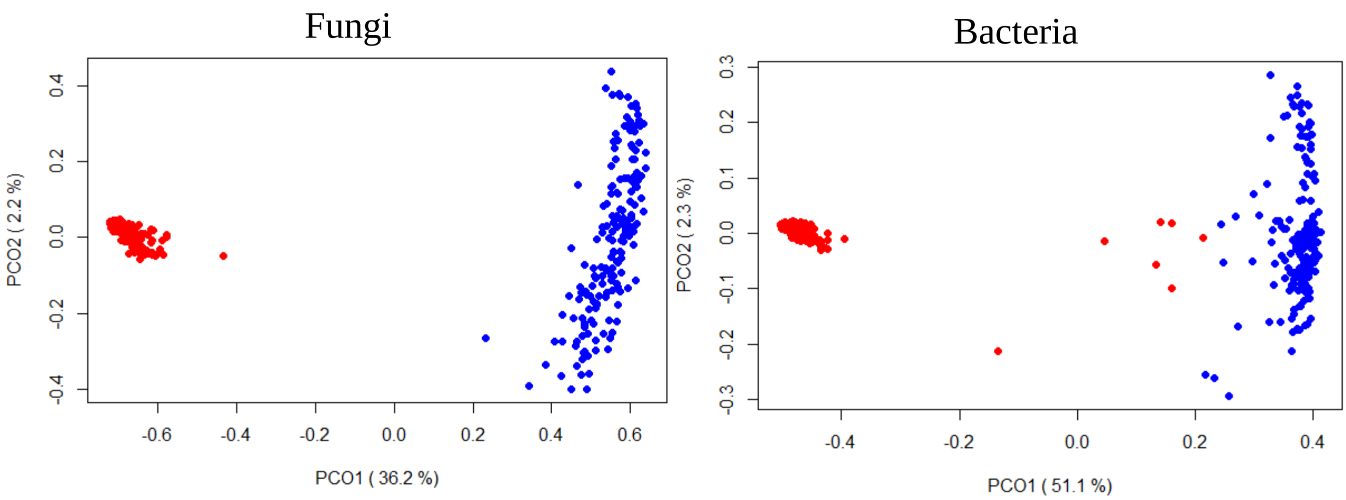

Table S4. Results of the permutational analyses of variance for both countries together, showing F-value, R^2, p-value, and significance of the considered factors.

P-values are significant at α = 0.1; · (P < 0.1), * (P < 0.05), ** (P < 0.01), *** (P < 0.001).

	F_fungi	*R2_fungi*	*Pr(>F)_fungi*	*Fungi*	*F. bact*	*R2_bact*	*Pr(>F)_bact*	*Bact*
Country	646.81	0.65873	0.001	***	357.32	0.51927	0.001	***
Fertilizer	7.19	0.00732	0.002	**	6.83	0.00993	0.002	**
Mono vs mixtures	1.13	0.00115	0.273		1.1	0.00159	0.261	
Crop species number (2 / 4)	1.87	0.00191	0.142		1.53	0.00222	0.17	
Cereal	2.97	0.00302	0.057	.	1.79	0.0026	0.111	
Legume	2.44	0.00249	0.08	.	1.74	0.00252	0.115	
Superasterid herb	1.86	0.00189	0.118		1.12	0.00162	0.283	
Country x fertilizer	5.69	0.00579	0.003	**	7.08	0.01028	0.001	***
Country x (mono vs mix)	0.86	0.00087	0.398		0.98	0.00143	0.325	
Country x crop species number	1.71	0.00175	0.153		1.34	0.00195	0.206	
Fertilizer x (mono vs mix)	0.91	0.00092	0.364		1.14	0.00166	0.259	
Fertilizer x crop species number	1.1	0.00112	0.279		0.87	0.00127	0.378	
Country x cereal	2.75	0.0028	0.054	.	1.76	0.00256	0.121	
Country x legume	2.12	0.00215	0.089	.	1.55	0.00226	0.138	
Country x superasterid herb	1.23	0.00125	0.25		1.27	0.00185	0.217	
Fertilizer x cereal	1.3	0.00132	0.238		1.28	0.00186	0.222	
Fertilizer x legume	1.02	0.00104	0.301		1.33	0.00193	0.187	
Fertilizer x superasterid herb	1.12	0.00114	0.265		1.09	0.00158	0.276	
Country x fertilizer x (mono vs mix)	0.96	0.00097	0.319		1.08	0.00157	0.289	
Country x fertilizer x crop species number	0.88	0.00089	0.388		0.93	0.00135	0.355	

Figure S3: Taxonomical abundance graph showing the relative proportion of the most abundant phyla of fungi (a) and bacteria (b).

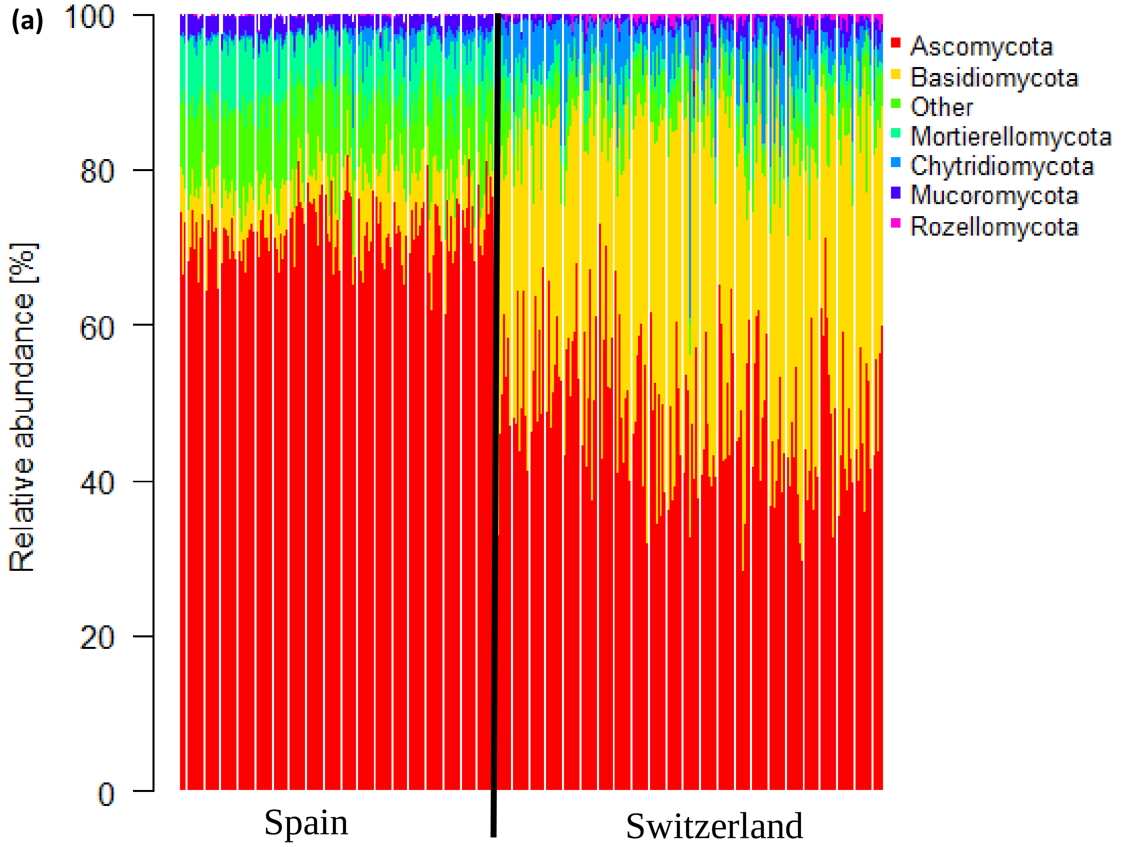

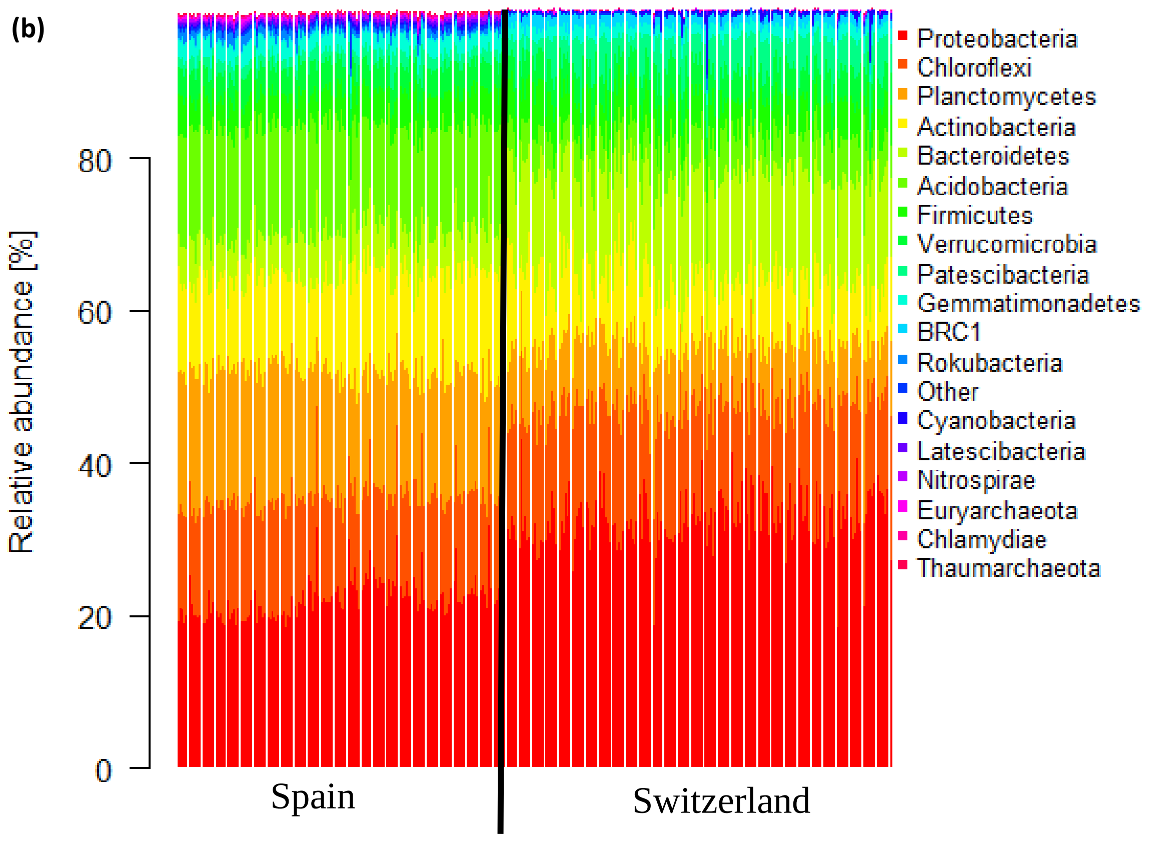

Figure S4: Differential abundance graph of fungal (a) and bacterial (b) phyla in the two different countries (Spain vs Switzerland).

A: percent abundance; r: point-biserial correlation coefficient; P.bh: Benjamini-Hochberg-corrected p-value.

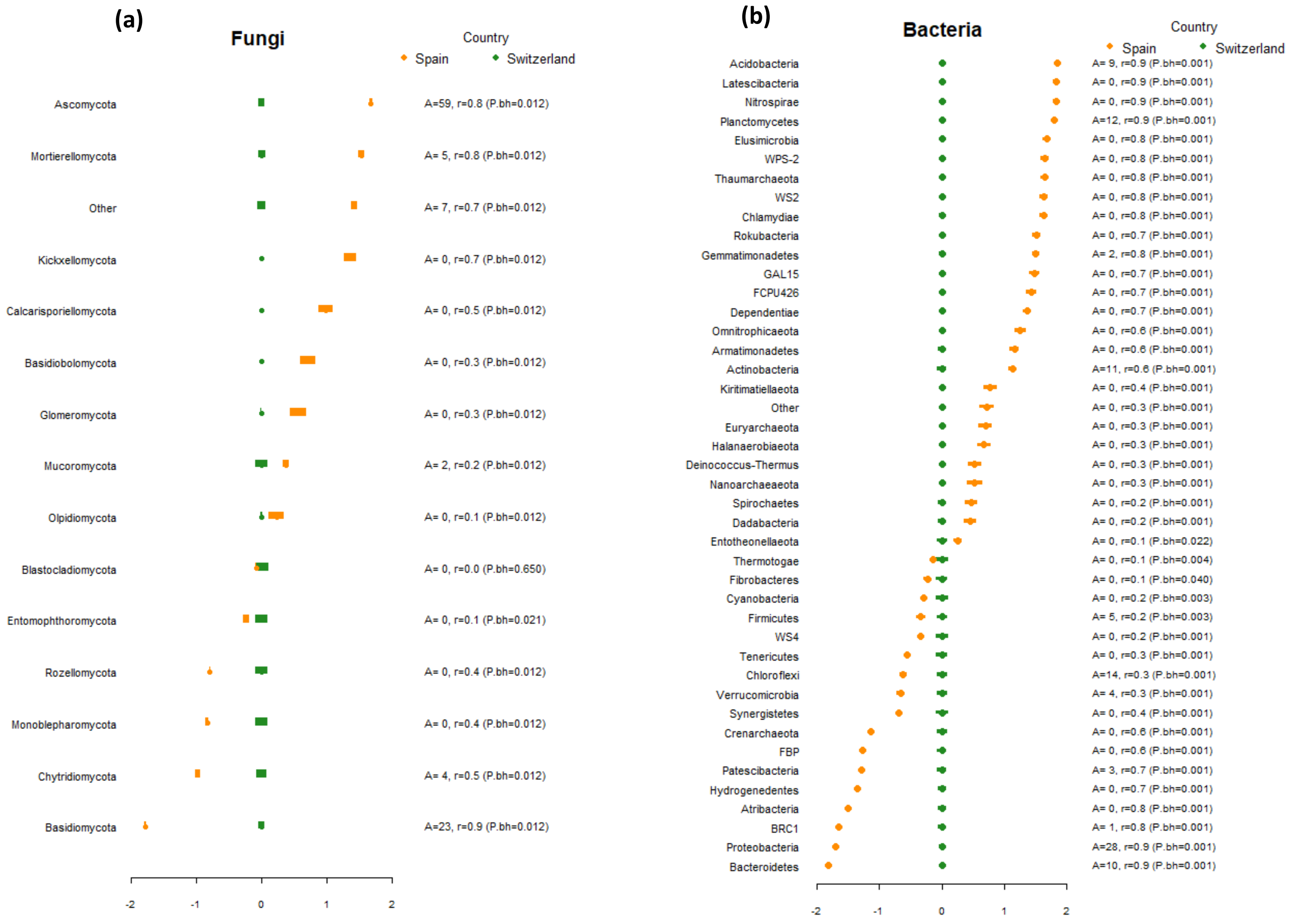

Figure S5. Constrained ordination plots showing changes in fungal communities in response to fertilizer (first line), cereal (second line), and crop species number (third line) in Spain (left panel) and in Switzerland (right panel). The circles represent the standard deviation of point scores of the ordination.

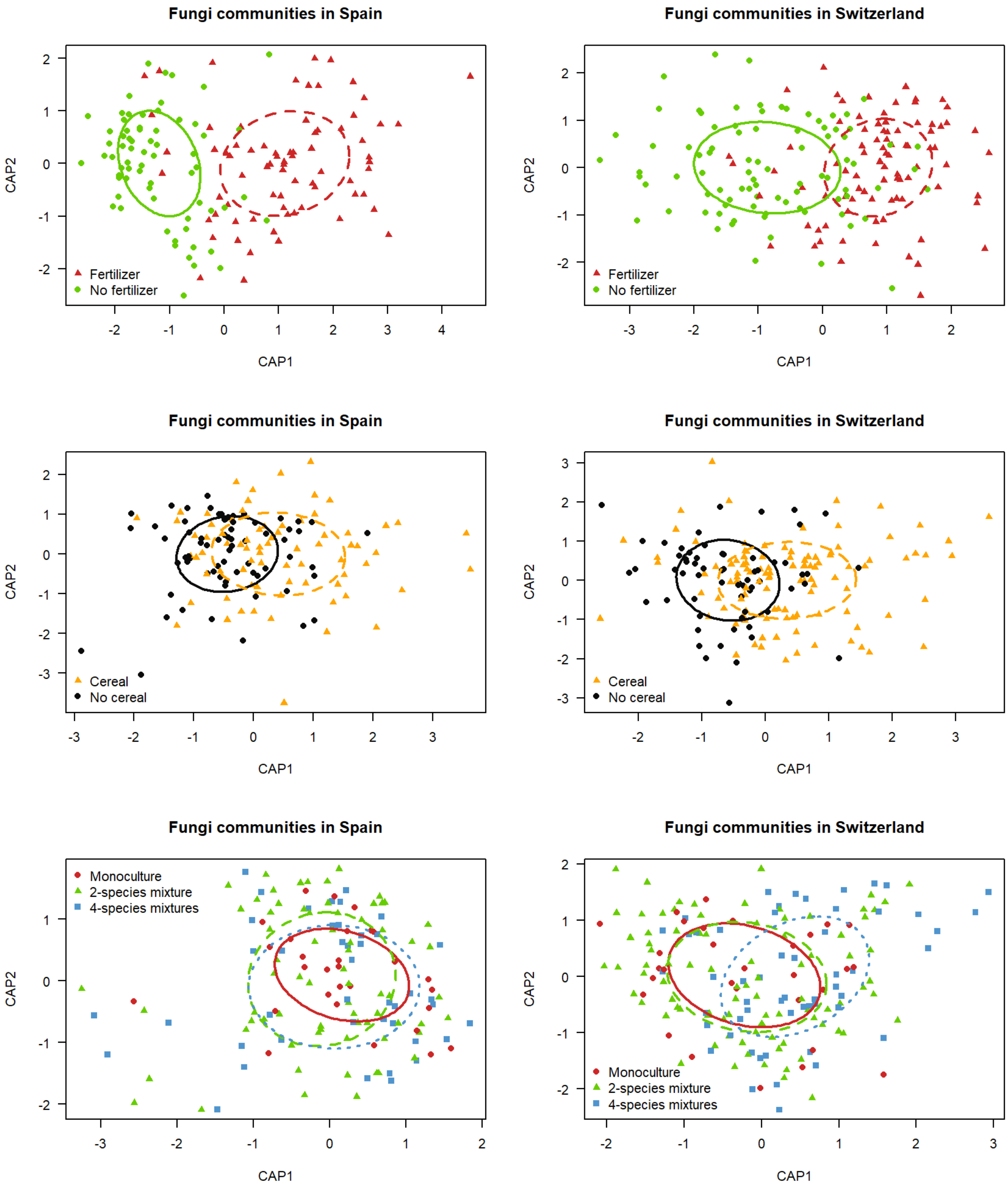

Figure S6: Constrained ordination plots showing changes in bacterial communities in response to fertilizer (first line), cereal (second line), and crop species number (third line) in Spain (left panel) and in Switzerland (right panel). The circles represent the standard deviation of point scores of the ordination.

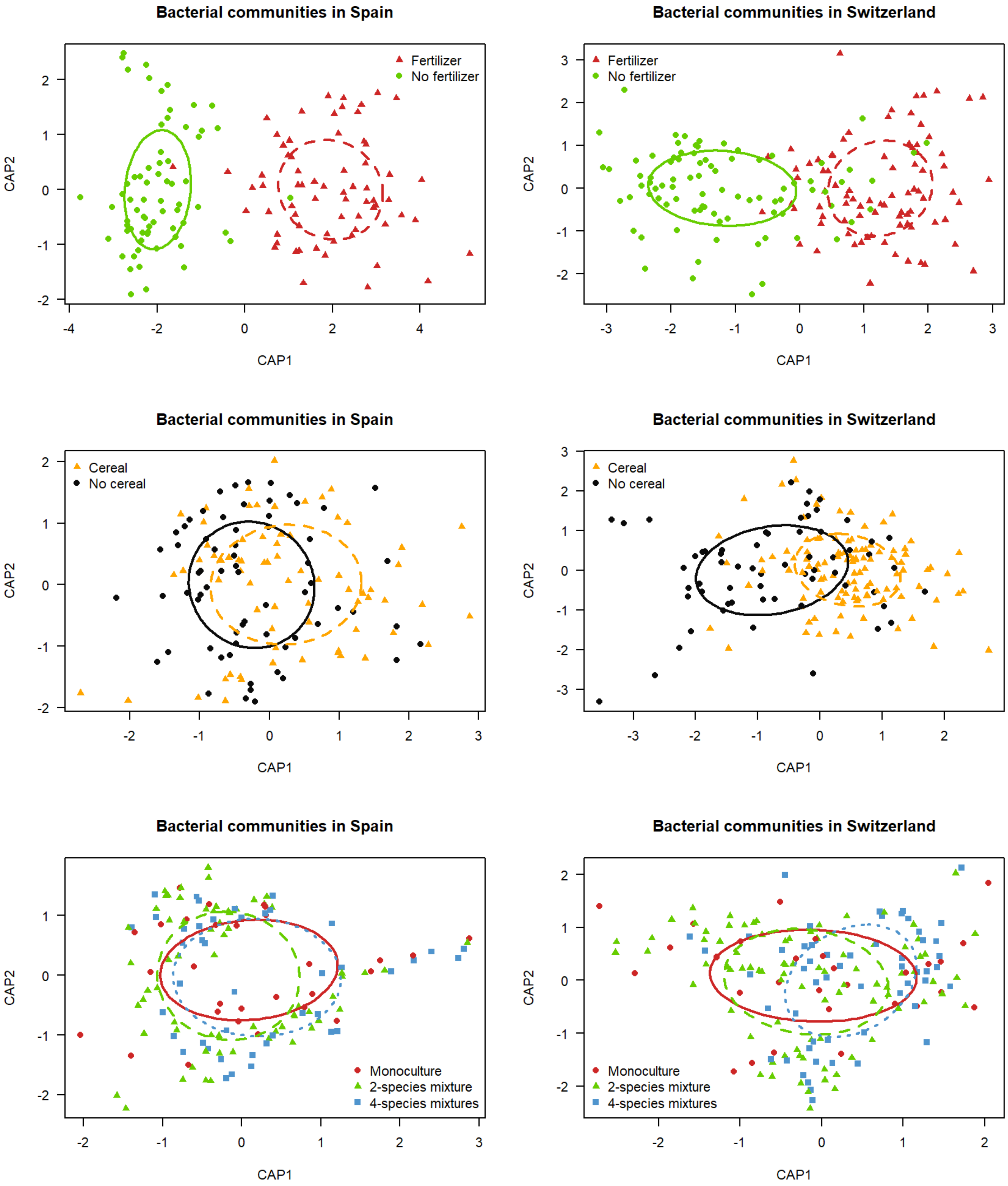

Figure S7: Differential abundance graph of fungi phyla under different fertilizing conditions in Spain (a) and Switzerland (b), and with or without cereal in Spain (c) and Switzerland (d).

A: percent abundance; r: point-biserial correlation coefficient; P.bh: Benjamini-Hochberg-corrected p-value.

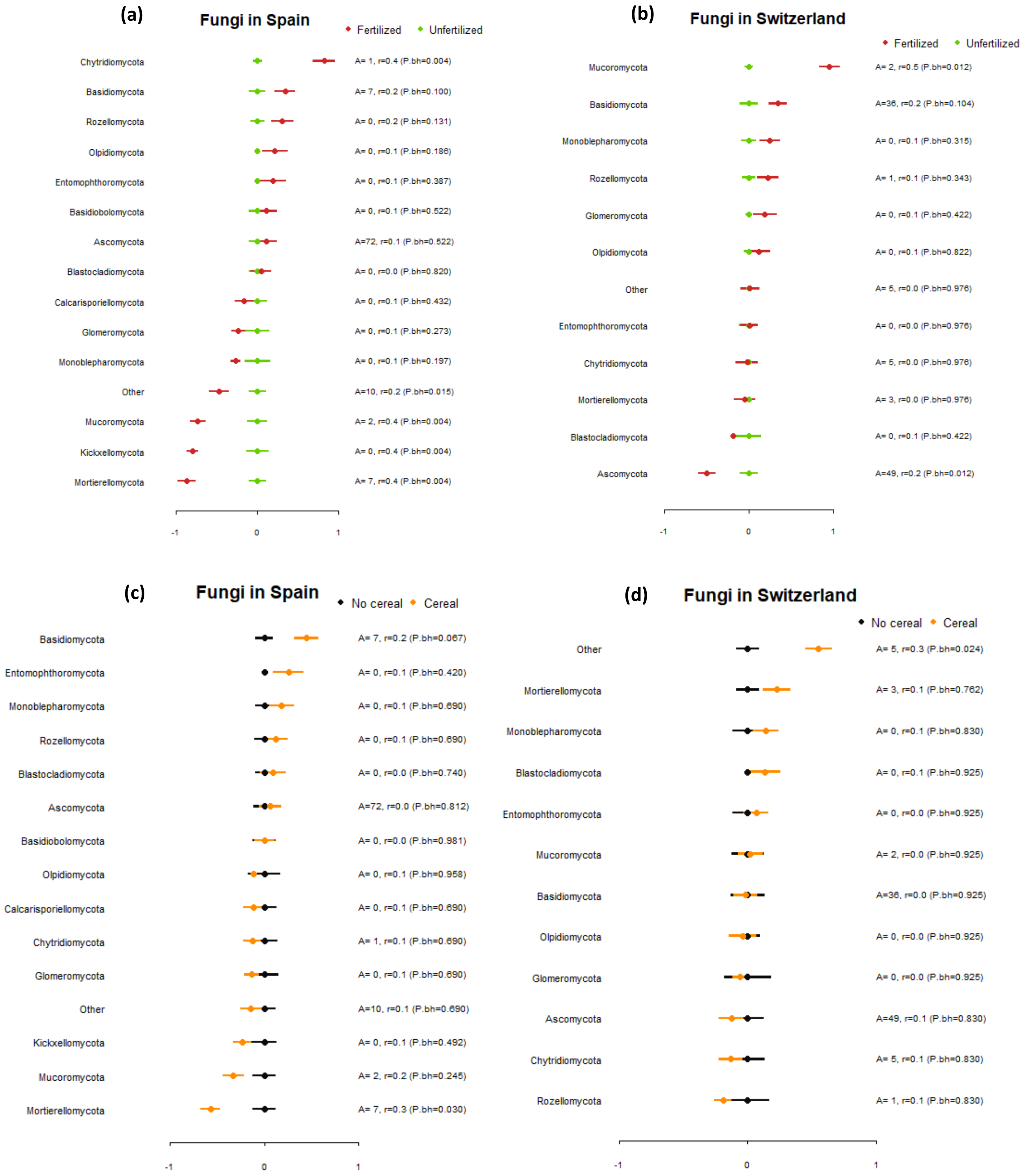

Figure S8: Differential abundance graph of bacterial phyla under different fertilizing conditions in Spain (a) and Switzerland (b).

A: percent abundance; r: point-biserial correlation coefficient; P.bh: Benjamini-Hochberg-corrected p-value.

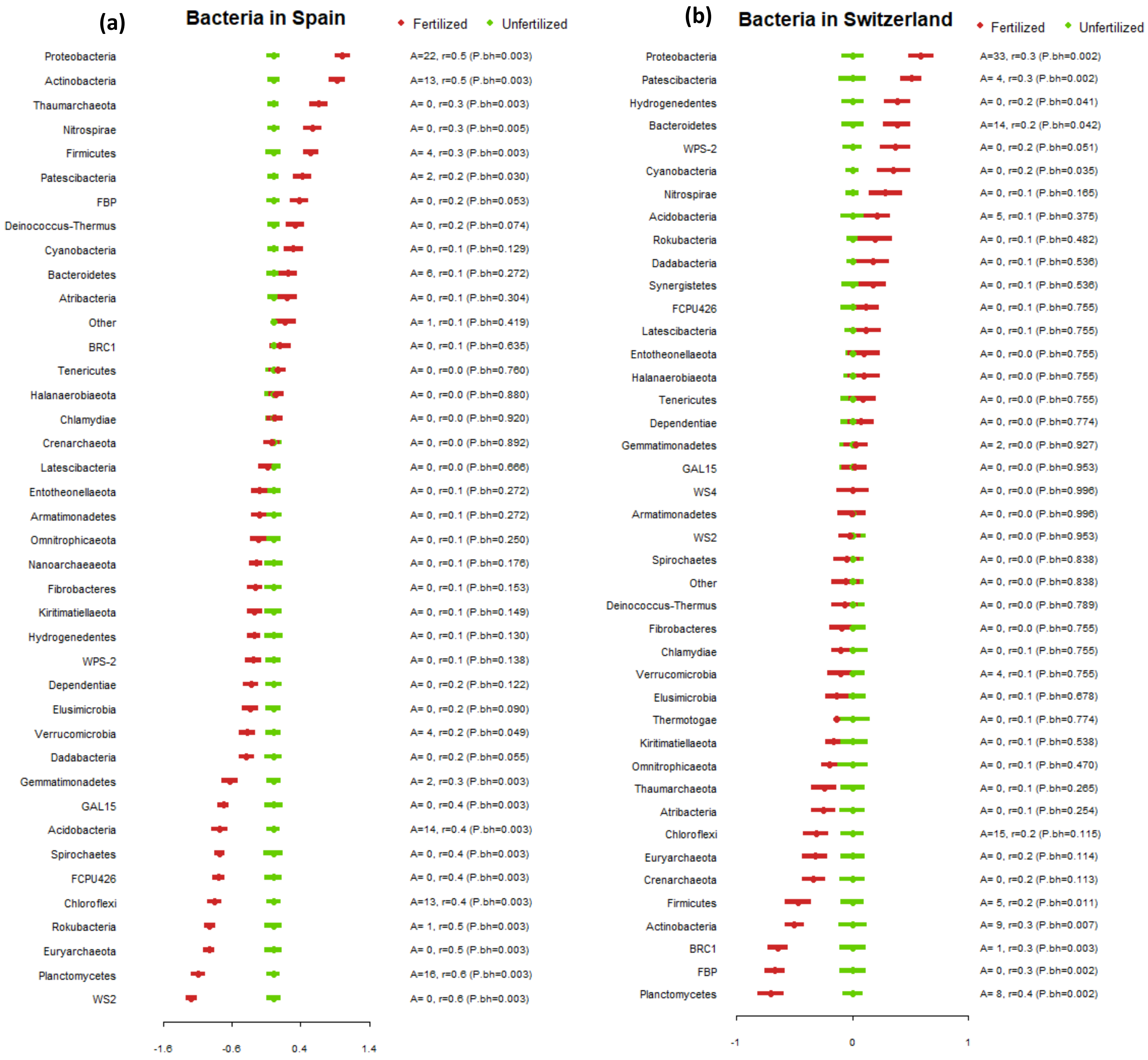

Figure S9: Differential abundance graph of bacterial phyla in Switzerland with or without cereal in Spain (a) and Switzerland (b).

A: percent abundance; r: point-biserial correlation coefficient; P.bh: Benjamini-Hochberg-corrected p-value.

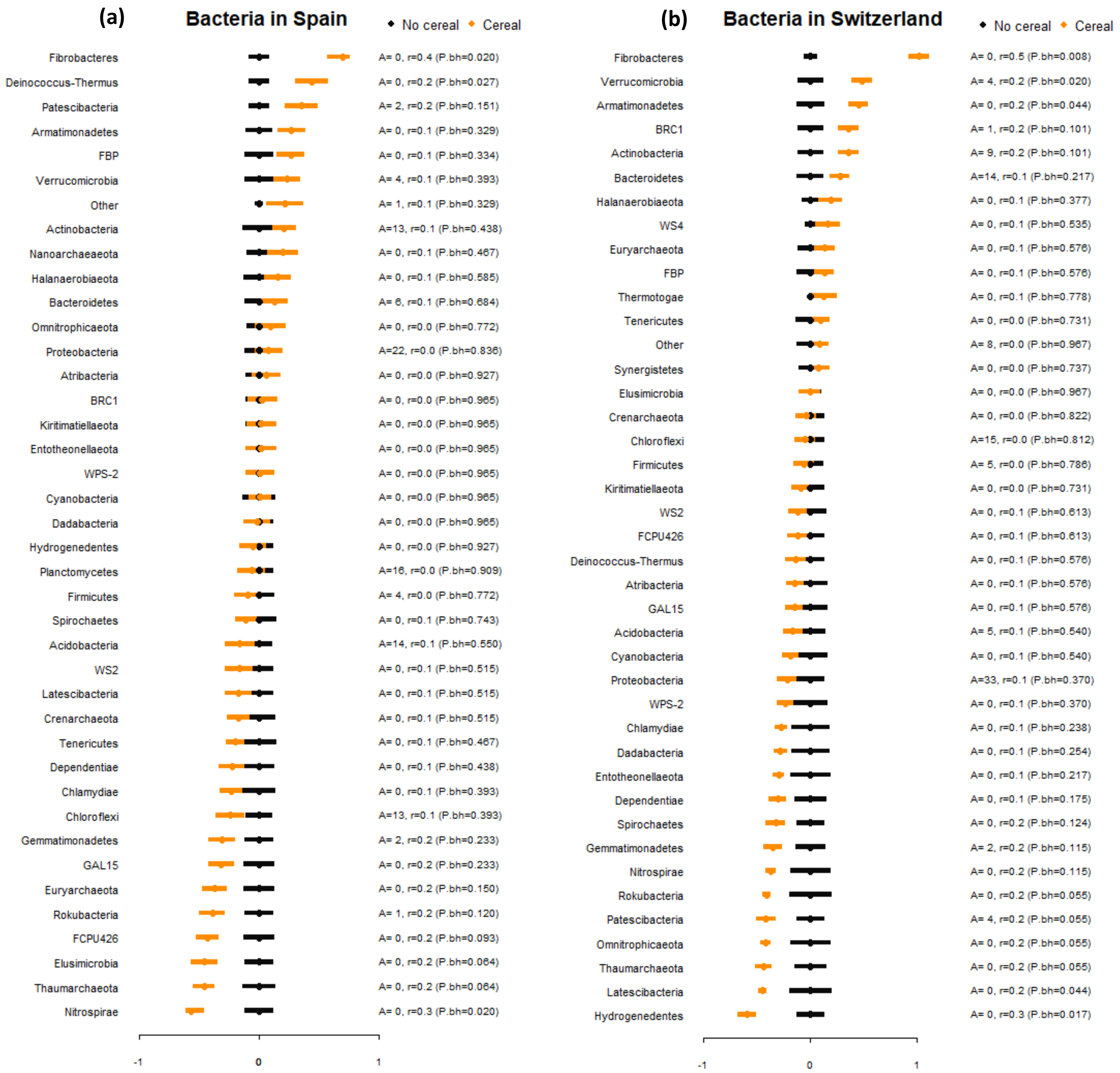

Table S5. Results of mixed effects ANOVA testing fertilizer, crop species number (i.e. two or four crop species) nested in monoculture vs. mixtures, and functional group presence on total crop yield in Spain. *DenDF*, degrees of freedom of error term; *NumDF*, degrees of freedom of term; *F-value*, variance ratio; *Pr(>F)*, error probability. P-values in bold are significant at α = 0.05; * (P < 0.05), ** (P < 0.01), *** (P < 0.001).

	NumDF	*DenDF*	*F value*	*Pr(>F)*
Fertilizer	1	12.314	4.1605	0.06343 .
Monoculture vs mixtures	1	37.596	0.1896	0.6657
Crop species number	1	41.585	0.1618	0.68957
Cereal	1	41.287	0.7085	0.40478
Legume	1	39.013	19.223	**8.53E-05 *****
Superasterrid herb	1	38.524	2.8635	0.09869 .
Fertilizer:mono. vs mix	1	91.759	0.0551	0.81487
Fertilizer:crop species number	1	98.888	2.7884	0.09811 .

Table S6. Results of mixed effects ANOVA testing fertilizer, crop species number (i.e. two or four crop species) nested in monoculture vs. mixtures, and functional group presence on total crop yield in Switzerland. *DenDF*, degrees of freedom of error term; *NumDF*, degrees of freedom of term; *F-value*, variance ratio; *Pr(>F)*, error probability. P-values in bold are significant at α = 0.05; * (P < 0.05), ** (P < 0.01), *** (P < 0.001).

	NumDF	*DenDF*	*F value*	*Pr(>F)*
Fertilizer	1	6.609	0.518	0.496353
Monoculture vs mixtures	1	41.593	7.989	**0.007203 ****
Crop species number	1	41.847	4.2773	**0.044842 ***
Cereal	1	41.738	5.5164	**0.023651 ***
Legume	1	41.749	7.3928	**9.50E-03 ****
Superasterrid herb	1	42.151	5.3418	**0.025779 ***
Fertilizer:mono. vs mix	1	113.712	1.4499	0.23105
Fertilizer:crop species number	1	106.636	0.0135	0.907629

Table S7. Results of mixed effects ANOVA testing fertilizer, crop species number (i.e. two or four crop species), and functional group presence on net biodiversity effect in Spain.
DenDF, degrees of freedom of error term; *NumDF*, degrees of freedom of term; *F-value*, variance ratio; *Pr(>F)*, error probability. P-values in bold are significant at α = 0.05; * (P < 0.05), ** (P < 0.01), *** (P < 0.001).

	NumDF	*DenDF*	*F value*	*Pr(>F)*
Fertilizer	1	12.392	0.002	0.964729
Crop species number	1	32.983	0.2269	0.636993
Cereal	1	31.56	15.3137	**0.000454 *****
Legume	1	32.453	7.0564	**0.012149 ***
Superasterrid herb	1	31.766	0.915	3.46E-01
Fertilizer:crop species number	1	12.392	0.002	0.964729 .

Table S8. Results of mixed effects ANOVA testing fertilizer, crop species number (i.e. two or four crop species), and functional group presence on net biodiversity effect in Switzerland.
DenDF, degrees of freedom of error term; *NumDF*, degrees of freedom of term; *F-value*, variance ratio; *Pr(>F)*, error probability. P-values in bold are significant at α = 0.05; * (P < 0.05), ** (P < 0.01), *** (P < 0.001).

	NumDF	*DenDF*	*F value*	*Pr(>F)*
Fertilizer	1	96.404	0.3049	0.582094
Crop species number	1	34.306	10.865	**0.002285 ****
Cereal	1	33.369	0.7424	0.395028
Legume	1	33.535	1.8868	0.178673
Superasterrid herb	1	33.308	0.2003	6.57E-01
Fertilizer:crop species number	1	96.404	0.3049	0.582094

Figure S10. Non-randomly distributed taxa along the second (a) and third (b) axes of fungal PCoA decomposition in Switzerland.

CH: Switzerland.

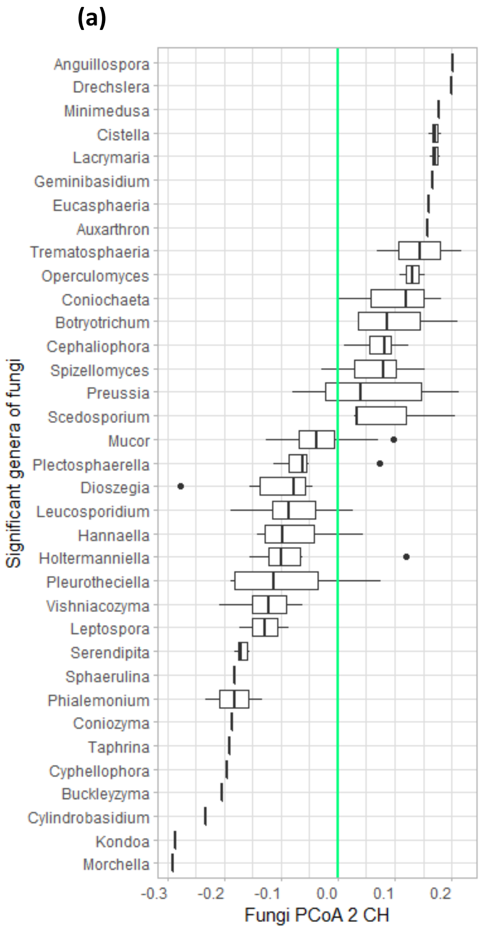

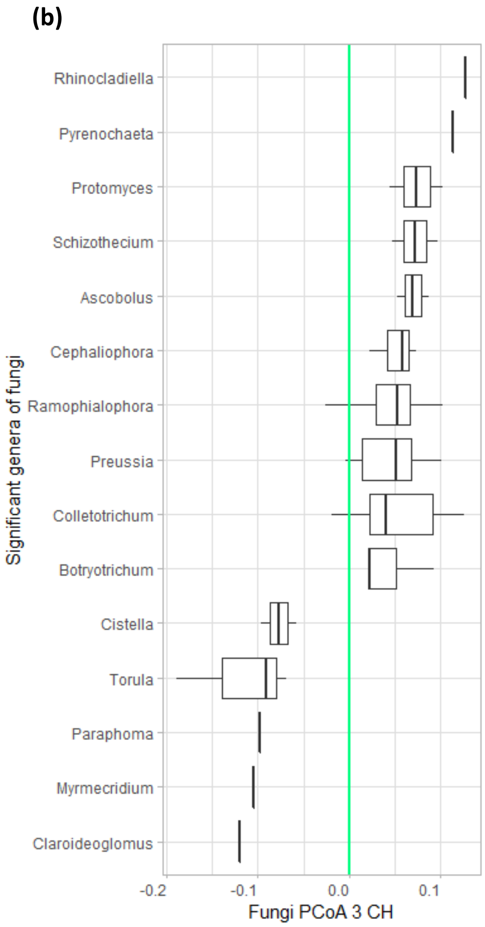

Figure S11. Non-randomly distributed taxa along the second axis of bacterial PCoA decomposition in Spain.

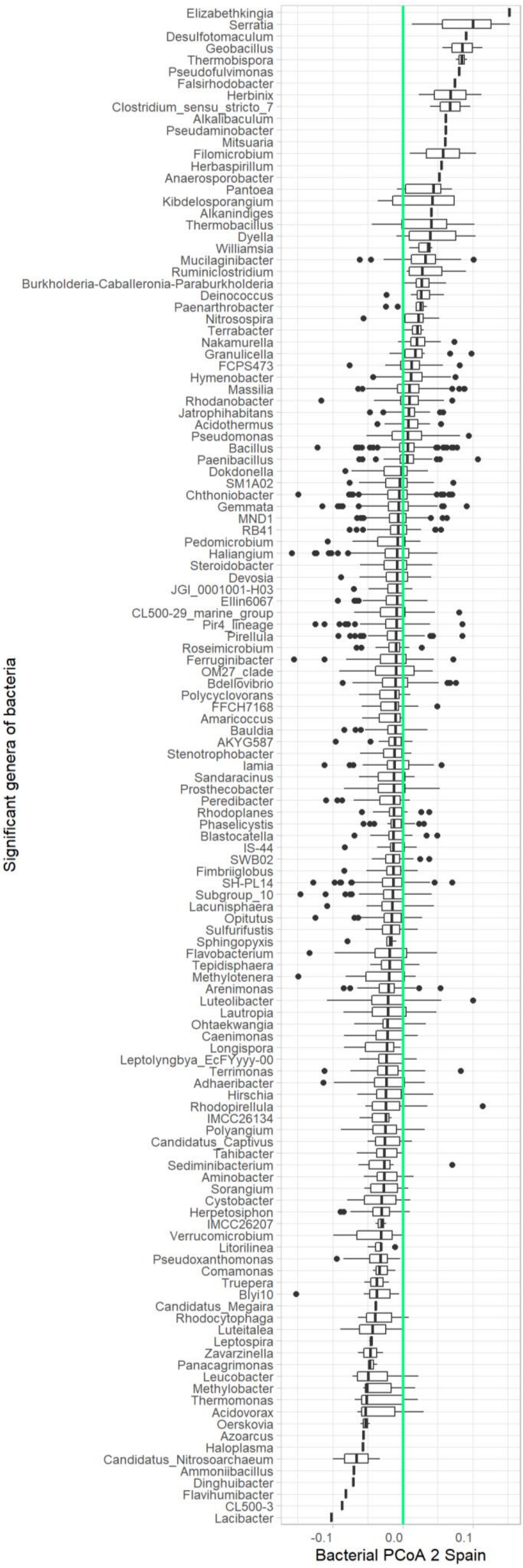

Figure S12. Non-randomly distributed taxa along the first (a) and second (b) axes of bacterial PCoA decomposition in Switzerland.

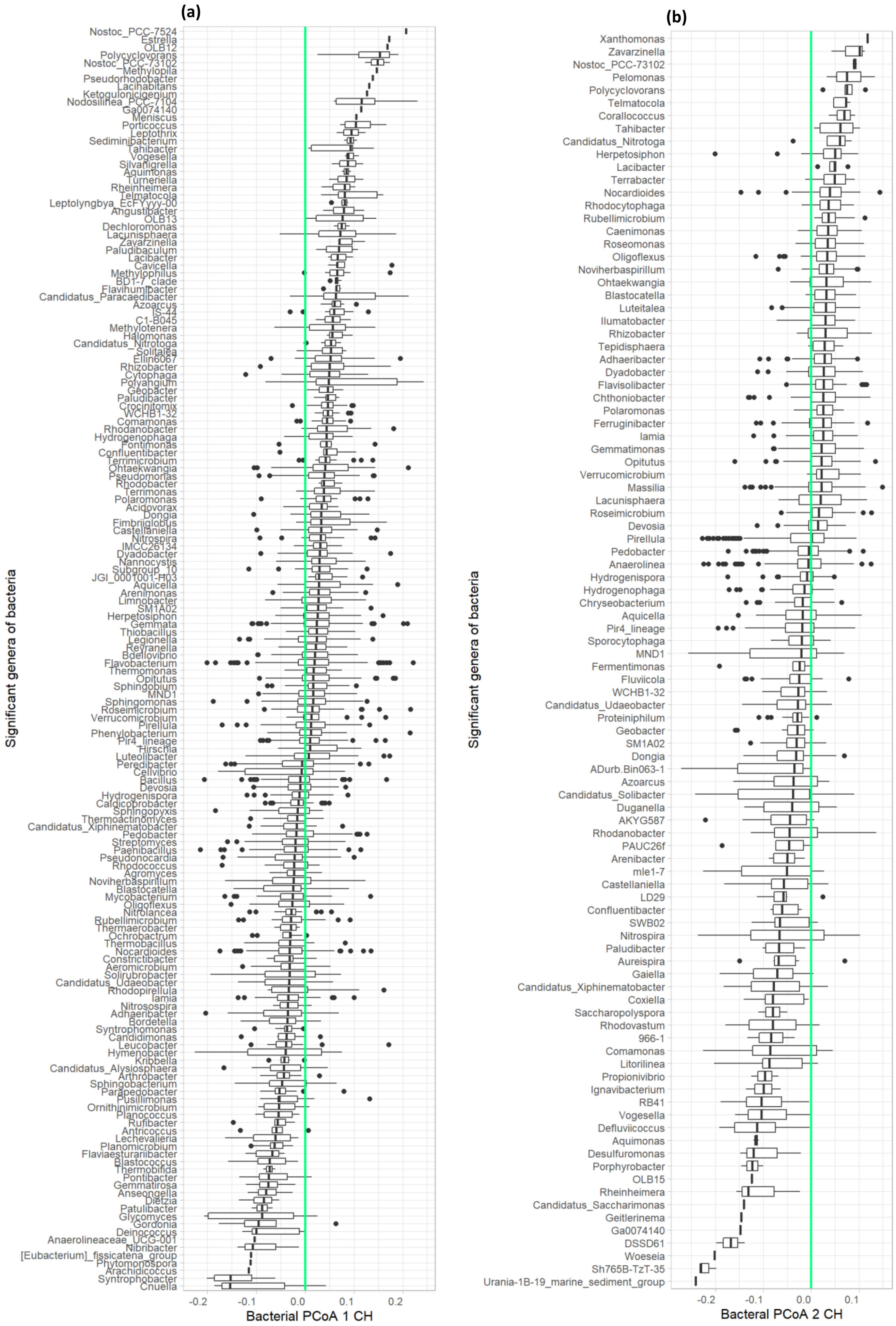

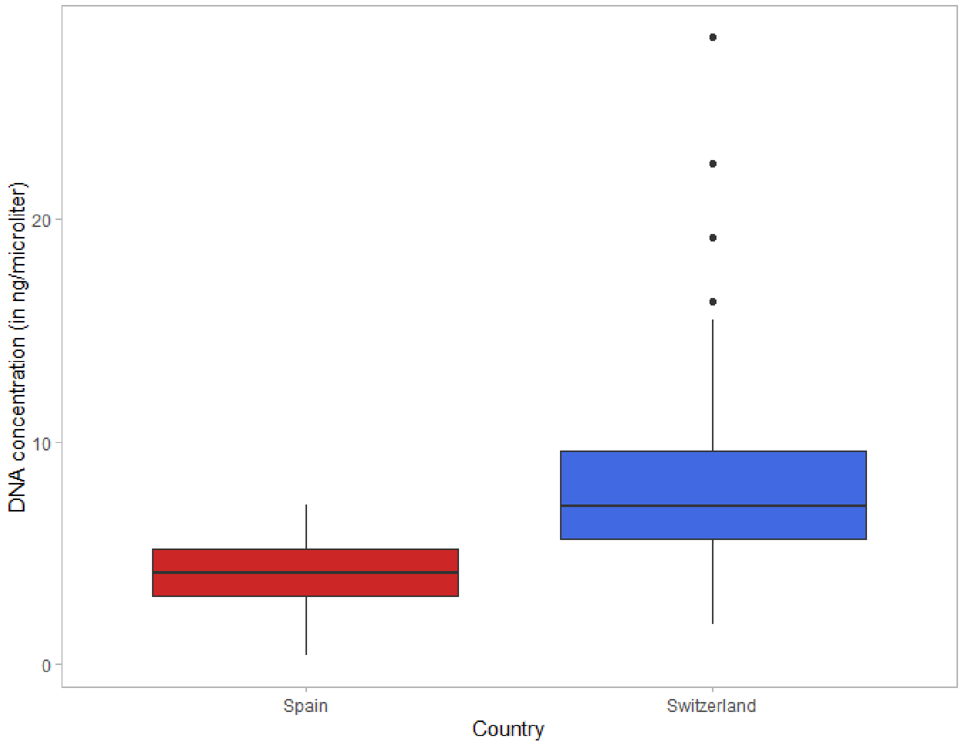

Figure S13. DNA concentration in ng/μL, in Spain and in Switzerland.
